# A new role of RAB21 and VARP in autophagy and autophagic exocytosis of ATP

**DOI:** 10.1080/27694127.2025.2501365

**Published:** 2025-05-11

**Authors:** María Carolina Barbosa, Pablo Reta, Sébastien Nola, Milton Osmar Aguilera, Thierry Galli, María Isabel Colombo, Claudio Marcelo Fader

**Affiliations:** a Laboratorio de Biología Celular y Molecular, Instituto de Histología y Embriología, (IHEM), Universidad Nacional de Cuyo, CONICET, Mendoza, Argentina; b Université Paris Cité, Institute of Psychiatry and Neuroscience of Paris (IPNP), INSERM U1266, Membrane Traffic in Healthy & Diseased Brain, Paris, France; c GHU PARIS psychiatrie & neurosciences, Paris, France; d Facultad de Odontología, Universidad Nacional de Cuyo, Mendoza, Argentina

**Keywords:** ATP release, LC3, macroautophagy, RAB proteins, secretory autophagy, VAMP7

## Abstract

Autophagy has been implicated in various cellular processes, including non-conventional secretion. Our previous findings suggest that ATP is loaded into amphisomes and secreted upon autophagy stimulation at focal adhesion sites in a VAMP7-dependent manner. Here, we demonstrate that the knockout (KO) of VAMP7, along with its partners RAB21 and its guanine nucleotide exchange factor (GEF) VARP, inhibits ATP release, indicating a key role for this pathway in amphisome secretion. Constitutively inactive RAB21 also inhibited ATP secretion. RAB21 overexpression rescued starvation-induced ATP secretion in RAB21 KO, but not in VAMP7 or VARP KO cells. RAB21-LC3-positive vesicles redistributed to the cell periphery upon starvation. KO cells and overexpression experiments showed that RAB21 plays a positive role in autophagosome biogenesis, particularly in controlling the number of LC3-II- and DFCP1-positive structures upon starvation, suggesting a role in the early steps of autophagosome formation. Accordingly, VARP partially colocalized with LC3 upon starvation. Together, these findings identify a novel role for RAB21 in regulating autophagic ATP secretion likely in amphisome biogenesis and their localization in the cell periphery.

## Introduction

Autophagy is considered one of the most important intracellular homeostatic processes [1,2]. Essentially, autophagy comprises three main lysosomal-based degradative pathways: microautophagy, chaperone-mediated autophagy and macroautophagy [2–4]. The last one, hereafter termed as autophagy, involves the engulfment of cytoplasmic components into a double membrane vesicle [1,2]. The initial step of autophagy consists of the recruitment of several proteins (such as ATG13, ATG101, ULK1 and FIP200) to an ATG9-rich membrane called phagophore [1,2,5–9]. Then, PI3-kinase, Beclin1 and ATG14 participate in the following elongation of the phagophore [1,2,9–16]. Finally, the phagophore closure is accomplished by another complex formed by the WD-repeat proteins Interacting with PhosphatidylInositol protein (WIPI), Double-Fyve Containing Protein 1 (DFCP1), ATG16L, ATG12, ATG5, ATG7 and ATG10 [[1,2,17–20]]. The newly formed vesicle called autophagosome is decorated with the Microtubule-Associated Protein Light Chain 3 (MAPLC3, hereafter called LC3) [1]. LC3 protein is anchored in both the inner and outer membranes, serving as a reliable marker of this compartment [21]. Newly formed autophagic vesicles fuse with endosomes to form amphisomes and finally with lysosomes to degrade the cargoes and recycle engulfed molecular components [1,2].

For the transport and membrane fusion of autophagosomes, several, Soluble NSF (N-ethylmaleimide-sensitive factor) Attachment protein REceptor (SNARE) and small GTPases (termed RABs) have been implicated. Among them, the SNARE Vesicular-Associated Membrane Protein 7 (VAMP7/TI-VAMP) was shown to be involved in phagophore formation, fusion with lysosomes and amphisomes as well as lysosomes secretion [22–24]. Notably, VAMP7 participates in the autophagosome-lysosome fusion in the fruit fly, in which VAMP8 is absent and the autophagosome transport to the cell periphery in mammalian cells relies on VAMP7 [22,25].

The small GTPases RABs, which are master regulators of intracellular trafficking [[26]], have also been directly or indirectly related to autophagy. RAB1, RAB5, RAB7, RAB8, RAB9, RAB11, RAB23, RAB24, RAB25, RAB32, and RAB33b are essential for autophagy, and the function of most of them has been widely described [27]. Interestingly, RAB21, which has been involved in the early endocytic pathway and also colocalizes with markers of the Golgi Apparatus (GA) [28], is part of the VAMP7 molecular hub and regulates its transport [29,30]. Moreover, RAB21 depletion leads to an augmented autophagic flux due to activation of AMPK-ULK1 axis in HeLa cells subjected to amino acid–free medium [[31]]. Additionally, RAB21 has been implicated in regulating autophagosome-lysosome fusion after starvation stimulation in *Drosophila melanogaster* and HeLa cells [32].

VAMP7 and RAB21 are connected at the molecular level by the guanine nucleotide exchange factors (GEF) of RAB21, VPS9 and Ankyrin Repeat Domain Protein (VARP), a multitasking protein ubiquitously expressed in mammals that also participates in endocytosis and exocytosis [23,30,33,34], RAB21 has been involved in multiple vesicular post-GA transport events as well as endosome-lysosome fusion, together with the v-SNARE VAMP8 in mammalian cells [28,32,35–37]. RAB21 associates with VAMP7 for the transport of endosomes and GA-derived vesicles to the cell periphery [34]. Notably, VARP silencing in HeLa cells led to a decline in LC3-positive dots compared with scramble knockdown in basal conditions, suggesting a role in the early steps of autophagy [32]. In yeasts, VARP-like 1 (Vrl1), a protein closely related to human VARP, plays an important role in autophagy under both basal and starvation conditions. Particularly, Vrl1 colocalized with Atg8 (the yeast homolog for LC3) after starvation in yeast mutants with defects in later steps of autophagy but not in wild type (WT) cells [38]. In addition, VARP and KIF5 interact at the molecular level [30] and KIF5 was shown to be involved in ATP secretion [22].

The data generated during the last decade expanded the autophagy function to a variety of other processes than degradation and recycling of cellular components. Secretory autophagy is considered one of the unconventional secretion types [39]. Proteins without any signal peptide cannot enter the conventional secretory pathway, which involves the translocation of the target protein into the endoplasmic reticulum (ER), the corresponding traffic to the GA and finally the fusion of the secretory vesicle with the plasma membrane [40]. Instead, leaderless proteins can be secreted in a nonconventional way, i.e., directly from cytoplasmic vesicles that fuse with the plasma membrane [41]. Furthermore, several proteins are secreted by autophagy or autophagy-related machinery [22,42–49]. Of our interest, Adenosine Triphosphate (ATP) has been reported to be secreted by several mechanisms to the extracellular space [50], including non-conventional autophagy-related exocytosis [51–53]. Extracellular ATP (eATP) can trigger numerous autocrine and paracrine responses [50,54], including the immune response, Immunogenic Cell Death (ICD), and aggressiveness phenotypes of several tumor cells [46,52,53,55,56]. Previous data from our laboratory demonstrate that autophagy stimulation induces the fusion of ATP-loaded amphisomes with the plasma membrane, releasing the ATP to the extracellular space in HeLa cells. Importantly, this fusion event is mediated by VAMP7 [22,25].

Our present report aims to provide evidence that RAB21 participates in early steps during autophagosome biogenesis and ATP autophagy-dependent secretion in HeLa cells. We first found that knocking out VAMP7, or its partners VARP and RAB21, inhibits ATP release, indicating a key role for this pathway in amphisome secretion. Accordingly, overexpression of constitutively inactive RAB21 mutant inhibited eATP after autophagy stimulation, further suggesting a central role of RAB21 in the ATP autophagy-dependent release. Moreover, overexpression of WT RAB21 modified the proportion of autolysosomes-autophagosomes at basal and starvation conditions, an effect which was abolished in RAB21 KO cells. Additionally, the overexpression of WT RAB21 leads to an increase in DFCP1-positive vesicles, suggesting a role for RAB21 in the phagophore closure. Notably, we found that starvation stimulus caused the delivery of RAB21 and VARP-labeled autophagosomes toward the cell periphery, possibly enabling them to fuse with the plasma membrane. Together, these results show a new role for RAB21 in the autophagy secretion of ATP, possibly at several steps of this process.

## Results

### RAB21, VARP, VAMP7 and KIF5A are necessary for autophagic ATP release in HeLa cells

**Figure 1.**
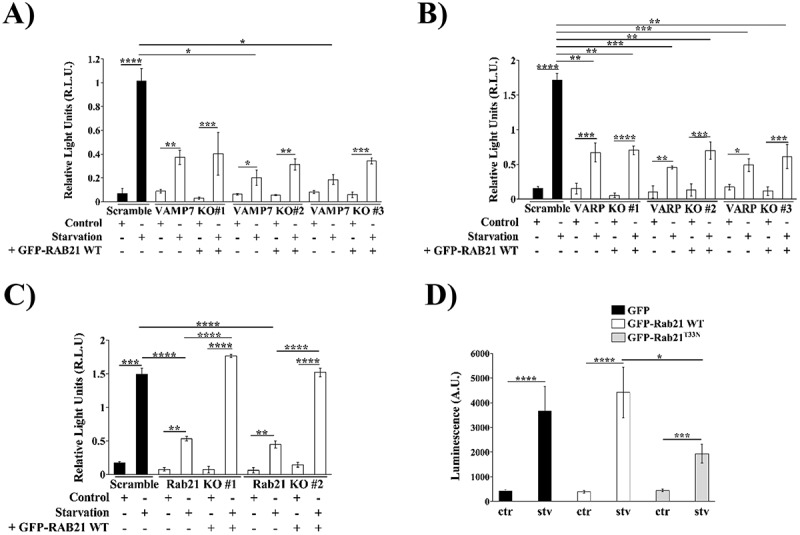
VAMP7, VARP and RAB21 are involved in the autophagic exocytosis of ATP in HeLa cells. VAMP7, VARP, RAB21 or scramble KO HeLa cells were incubated in control or starvation conditions for 2 hours. Medium samples were taken to conduct a Luciferin-Luciferase assay (A) luminescence measurements of scramble or VAMP7 KO cells. A subpopulation of VAMP7 KO cells were transfected with GFP-RAB21 WT for phenotype recovery assessment. Luminescence is expressed in relative light units (R.L.U.). (B) Luminescence measurements of scramble or VARP KO cells. A subpopulation of VARP KO cells were transfected with GFP-RAB21 WT for phenotype recovery assessment. Luminescence is expressed in relative light units (R.L.U.). (C) Luminescence measurements of scramble or RAB21 KO cells. A subpopulation of RAB21 KO cells were transfected with GFP-RAB21 WT for phenotype recovery assessment. Luminescence is expressed in relative light units (R.L.U.). (D) Luminescence measurements of HeLa cells overexpressing GFP, GFP-RAB21 WT or GFP-RAB21^T33N^ that were exposed to control (ctr) or starvation (stv) conditions for 2 hours. Luminescence is expressed in arbitrary units (A.U.). All the graphs show the mean ± SEM of at least three independent experiments. Statistical analyses were done using raw or transformed data and a 2-way ANOVA was performed. Significance: *p ≤ 0.05; **p ≤ 0.01; ***p ≤ 0.001 and ****p ≤ 0.0001.

Because VAMP7 was previously proposed to mediate ATP-loaded vesicles transport to the cell periphery [22,25], we wondered if its KO could impair ATP secretion. Scramble KO or VAMP7 KO cells were incubated in control or starvation conditions for 2 hours, and then a Luciferine-Luciferase assay was carried out. VAMP7 KO cells exhibited a significant decrease in eATP compared to scrambled cells (Figure 1(A) and Fig. S1A and D). RAB21 and VARP have been previously shown to be involved in the delivery of VAMP7 endosomes from the cell center to the cell periphery [29,57]. Thus we decided to evaluate their implication in ATP secretion. Both RAB21 and VARP KO HeLa cells showed a significant decrease in eATP in comparison with scrambled cells (Figure 1(B,C) and Fig. S1B and E-H). Due to the fact that VARP and VAMP7 interact with KIF5A [30], and previous results demonstrated that KIF5A interacting domain (ID) overexpression results in a decrease in VAMP7-positive structures at the cell periphery [22] and because ID dominant negative effect has been previously reported [30], we performed a bioluminescence detection of eATP in HeLa cells overexpressing KIF5A WT or its ID. Transfected cells were then incubated in control or starvation for 2 hours. The results show that overexpression of the ID slightly decreased eATP in comparison with GFP-empty vector or GFP-KIF5A overexpressing cells, suggesting that KIF5A could be involved in autophagic exocytosis of ATP (Fig. S2), in agreement with previous results [22,30]. Altogether, these data suggested that the VAMP7 molecular network (VARP, RAB21, KIF5A) plays an important role in autophagic secretion of ATP.

To further unravel a role of RAB21’s activity in autophagy secretion of ATP, HeLa cells overexpressing the GFP-empty vector, GFP-RAB21 WT or GFP-RAB21^T33N^ were incubated in control or starvation conditions for 2 hours and a Luciferin-Luciferase assay was conducted to measure the eATP. The results show that the overexpression of RAB21^T33N^ significantly decreased the released eATP in comparison with GFP or RAB21 WT overexpressing cells after autophagic stimulation (Figure 1(D) and Fig. S1I-J), further supporting the involvement of RAB21 in the ATP autophagy-dependent exocytosis in HeLa cells and suggesting that its activity might be important. Furthermore, overexpression of GFP-RAB21 WT completely rescued the KO phenotype, suggesting an important role of RAB21 in ATP autophagy-dependent secretion (Figure 1(C) and Fig. S5C and D).

We then asked whether the positive effect of RAB21 overexpression on ATP secretion was still dependent on VARP and VAMP7. Indeed, this was the case as the overexpression of GFP-RAB21 WT did not affect the inhibition resulting from KO of VARP and VAMP7 in both control and starvation conditions (Figure 1(A,B) and Fig. S1D and E). This suggested that RAB21 played a central role in ATP secretion in a pathway depending on VAMP7 and VARP.

### Overexpression of RAB21 modifies the subcellular distribution of autophagosomes in starvation

**Figure 2.**
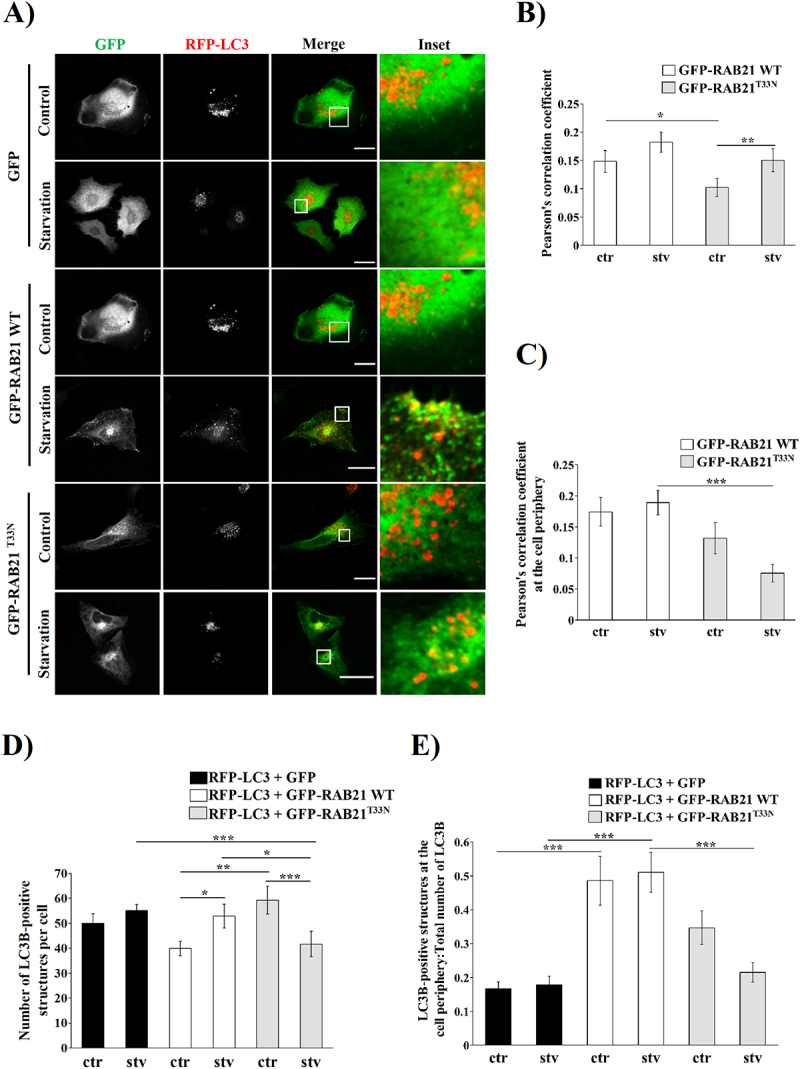
RAB21 colocalized with LC3-positive structures after autophagy stimulation. (A) Representative images of HeLa cells overexpressing GFP or GFP-RAB21 (WT or its constitutively inactive mutant RAB21^T33N^) and RFP-LC3 after control (ctr) or starvation (stv) incubation for 2 hours. Scale bar: 30 µm. Pearson’s correlation coefficient quantification at the whole cell (B) or at the cell periphery (C). (D) Quantification of number of LC3-positive structures at the whole cell of cells shown in (A). (E) Number of LC3-positive structures at the cell periphery, normalized to the whole cell quantification of each cell shown in (D). All the graphs show the mean ± SEM of at least three independent experiments. Statistical analyses were done using raw or transformed data and a 2-way ANOVA was performed. In cases where normality was not reached, non-parametric tests were used. Significance: *p ≤ 0.05, **p ≤ 0.01, ***p ≤ 0.001 and ****p ≤ 0.0001.

We then wondered if Rab21’s central role in autophagic secretion of ATP could be due to a role in autophagy per se. To address this question, we first analyzed the RAB21 subcellular distribution upon starvation-induced autophagy. HeLa cells overexpressing GFP-RAB21 WT or a constitutively inactive mutant, GFP-RAB21^T33N^, were co-transfected with RFP-LC3. Twenty-four hours later, the cells were incubated in full or free Fetal Bovine Serum (FBS) conditions (control and starvation media respectively) for 2 hours. The samples were processed for confocal microscopy, and representative images are shown in Figure 2(A). Colocalization analysis showed that the total amount of RAB21-LC3-positive structures was lower in cells expressing RAB21^T33N^ compared to WT both in control and starvation conditions (Figure 2(B)). Interestingly, when the colocalization was measured using a specific ROI at the cell boundary (Fig. S3), RAB21^T33N^ overexpressing cells showed a marked and significant decrease in colocalization with LC3 in starved cells (Figure 2(C)).

To further characterize the distribution of RAB21-positive structures after autophagy induction, we conducted a time-lapse assay in which HeLa cells were transfected with GFP-RAB21 WT or GFP-RAB21^T33N^ and incubated in control or starvation conditions for 2 hours. Then, live imaging was carried out for 10 minutes under the confocal microscope (Fig. S4A and B and Movies 01 and 02). Vesicle tracking analyses showed that GFP-RAB21 WT overexpressing cells displayed an increased proportion of vesicles that move toward the cell periphery both in control and starvation conditions in comparison to the GFP-RB21^T33N^ overexpressing cells (Fig. S4C). These results support the importance of an active RAB21 in its redistribution after starvation induction.

**Figure 3.**
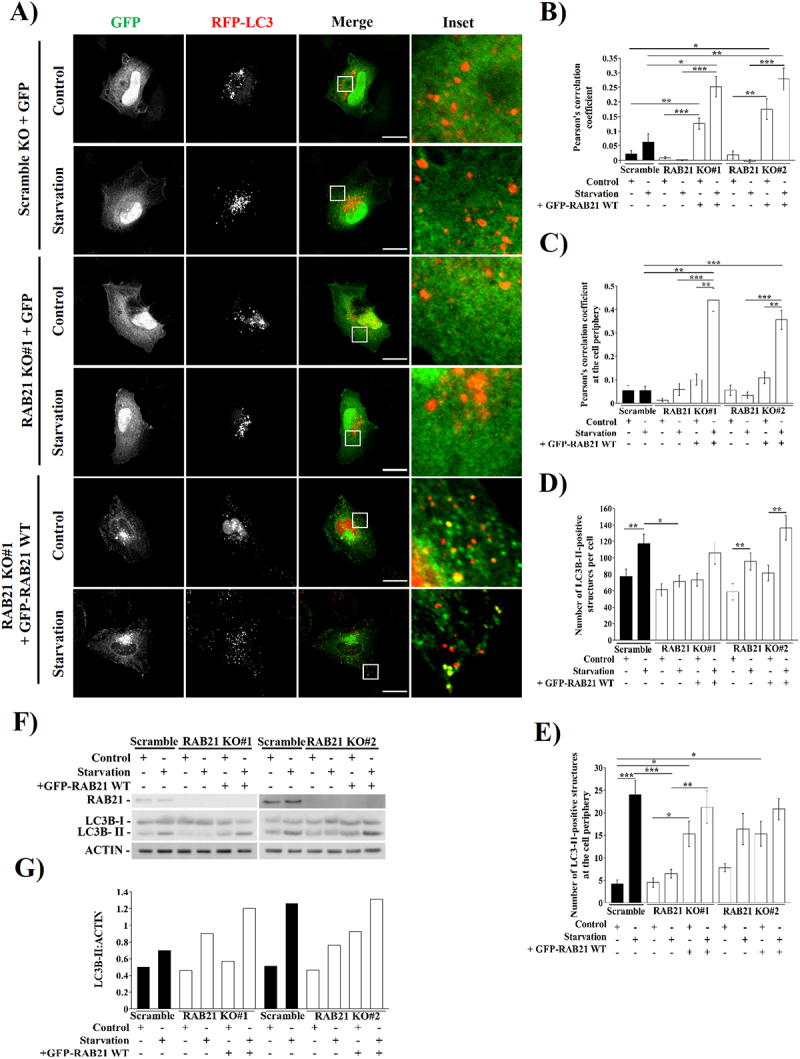
RAB21 KO HeLa cells abolishes the redistribution of LC3-positive structures to the cell periphery after autophagy stimulation. (A) Representative images of scramble or RAB21 KO#1 heLa cells overexpressing a RFP-LC3 construct co-transfected with GFP or GFP-RAB21 WT were incubated in control or starvation conditions for 2 hours. Similar images were obtained with RAB21KO#2 clones (data not shown). Scale bar: 30 µm. Pearson’s correlation coefficient quantification at the whole cell (B) or at the cell periphery (C). (D) Quantification of the number of LC3-positive structures of cells shown in (A). (E) Quantification of number of LC3-positive structures at the cell periphery of cells shown in (A). (F) Western Blotting of scramble or RAB21 KO cells where LC3B-II levels were examined. (G) Quantification of LC3B levels relative to ACTIN of the immunoblotting shown in (F). The graph shows the mean of two replicates. The resting graphs show the mean ± SEM of at least three independent experiments. Statistical analyses were done using raw or transformed data and a 2-way ANOVA was performed. In cases where normality was not reached, non-parametric tests were used. Significance: *p ≤ 0.05, **p ≤ 0.01 and ***p ≤ 0.001.

Regarding autophagic vesicles distribution, when LC3-positive structures were quantified, there was a significant decrease after starvation treatment in RAB21^T33N^ cells in comparison with WT (Figure 2(D)). Interestingly, GFP-RAB21 WT overexpressing cells had a significant increase of LC3-positive structures at the cell periphery in the starvation condition compared to the GFP or GFP-RAB21^T33N^ transfected cells (Figure 2(E)). Of note, even at full media condition, the total number of LC3 puncta at the cell periphery was significantly increased in comparison to GFP transfected cells (Figure 2(E)). Together, these results show that RAB21 partially colocalized with LC3, and this colocalization at the cell periphery relies on RAB21’s activity after autophagy stimulation. To confirm this, we transfected RFP-LC3 and GFP or GFP-RAB21 WT constructs in RAB21 KO cells (Figure 3(A)) and we incubated them in full or starvation media for 2 hours. Microscopy analyses showed an increase in GFP and RFP colocalization after starvation stimulus in RAB21 KO cells complemented with the GFP-RAB21 WT protein (Figure 3(B,C)). Interestingly, the number of LC3-II-positive structures per cell as well as at the cell periphery showed a decrease in RAB21 KO cells in comparison to the Scramble KO cells after starvation. Of note, GFP-RAB21 complementation successfully rescued the Scramble phenotype (Figure 3(D,E)), reinforcing the involvement of RAB21 in autophagy. We then measured the LC3B-II content in our two independent RAB21 KO clones incubated in control or starvation medium for 2 hours. The increase on the LC3B-II level was partially impaired in both RAB21 KO clones. Re-expressing RAB21 WT in KO cells restored the levels of LC3B-II similar to control levels both in full and starvation medium conditions (Figure 3(F,G)).

The differential colocalization between RAB21 and LC3 after starvation incubation led us to hypothesize that RAB21 May have an early role in autophagy. To further investigate the relationship between RAB21 and LC3, GFP-RAB21 WT was overexpressed in HeLa cells and we conducted a GFP-Trap assay in order to evaluate if the colocalization between RAB21 and LC3 was due to a direct interaction or a complex formation among the proteins. As shown in Fig. S5, we did not find any direct interaction of RAB21 with LC3, in agreement with the absence of a LIR motif in RAB21 searched *in silico* (data not shown [58]). This result suggests that RAB21 and LC3 could decorate the same autophagosomes, but they would not form any protein complex.

### Overexpression of RAB21 alters the autophagy flux, leading to an accumulation of non-acidic vesicles

**Figure 4.**
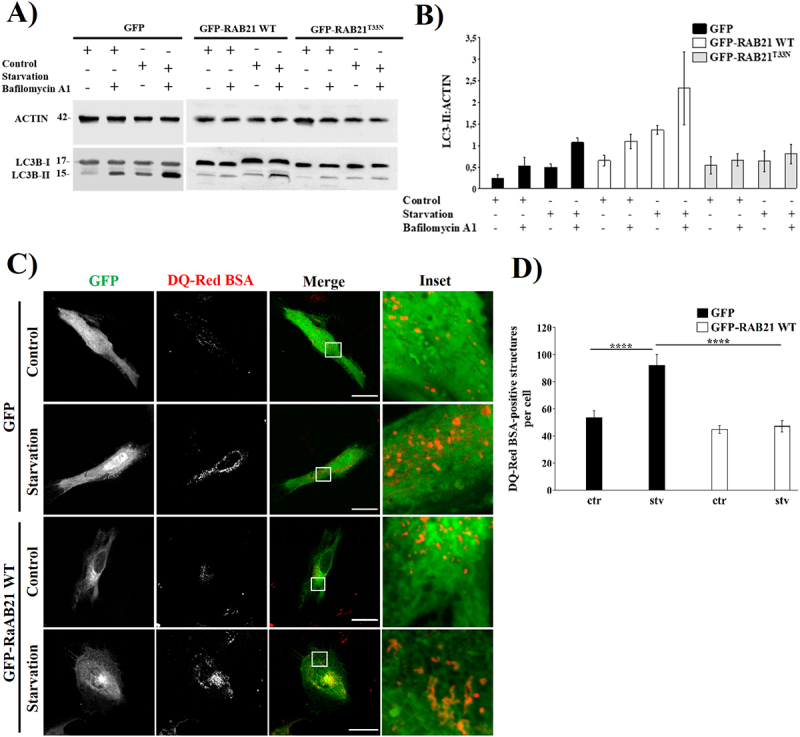
Overexpression of RAB21 WT alters the autophagy flux and leads to an increase of nonacidic autophagosomes. (A) A representative Western Blot of an autophagy flux assay of HeLa cells overexpressing GFP, GFP-RAB21 WT or GFP-RAB21^T33N^ incubated in control or starvation conditions for 2 hours in presence or absence of 100 nM bafilomycin A1. LC3 levels were examined and actin was used as loading control. (B) Quantification of LC3B-II:ACTIN of bands shown in (A). The graph shows the mean ± SEM of at least three independent experiments.Transformed data was used to conduct a three-way ANOVA and a *posteriori* multiple comparison test for statistical analyses. No statistical analyses were found. (C) DQ red BSA red internalization and degradation assay was conducted in HeLa cells overexpressing GFP or GFP-RAB21 WT incubated in control or starvation conditions for 2 hours. Representative images of each treatment are shown. Scale bar: 30 µm. (D) Quantification of DQ red BSA-positive structures per cell. The graph shows the mean ± SEM of at least three independent experiments and statistical analyses were performed using raw data and a 2-way ANOVA. Significance: ****p ≤ 0.0001.

The colocalization between RAB21 and LC3 raised new questions regarding the role of this RAB in autophagy. Because a significant decrease in colocalization between LC3 and RAB21 was found in GFP-RAB21^T33N^ overexpressing cells (Figure 2(B,C)), as well as the redistribution to the cell periphery of LC3-positive vesicles after GFP-RAB21 WT overexpression (Figure 2(E)), we used an autophagy flux assay in our cellular models. Autophagic flux is a measure of autophagic degradation activity. It is the whole process of cargo moving through the autophagy pathway, from phagophore formation to autophagosome-lysosome fusion and cargo degradation as well as its recycling. To evaluate if overexpression of RAB21 could alter the autophagic flux, GFP-RAB21 WT or GFP-RAB21^T33N^ overexpressed cells were incubated under control or starvation conditions, supplemented or not with Bafilomycin A1, a well-known autophagosome-lysosome blocker [59–61]. As depicted, in GFP-RAB21 WT overexpressing cells, an increase in the LC3B-II band was observed in cells subjected to control or starvation conditions either in the presence or not of Bafilomycin A1. In contrast, no such increase was observed in cells overexpressing GFP or GFP-RAB21^T33N^ (Figure 4(A,B)). Of interest, overexpression of RAB21^T33N^ abolishes the increase in LC3B-II band due to Bafilomycin A1, suggesting that overexpression of the RAB21 constitutively negative mutant may alter autophagy in cells subjected to starvation conditions (Figure 4(A,B)). To confirm this, we carried out a DQ Red BSA internalization-degradation experiment as another way to evaluate the autophagic flux. HeLa cells overexpressing GFP-RAB21 WT or GFP were incubated for 2 hours in control or starvation medium containing DQ Red BSA (a marker of the lysosomal degradative compartment). As shown in Figure 4(C,D), overexpression of RAB21 WT decreased the number of DQ Red BSA-positive dots compared to the GFP. These results suggest that overexpression of RAB21 WT decreased the number of degradative structures in both control and starvation conditions.

Another strategy to examine the autophagic flux is the overexpression of a tandem LC3 (mCherry-GFP-LC3B). In the autophagosome, this probe presents a double fluorescence emission provided by GFP and mCherry that combined leads to an overall yellow fluorescence signal when the two fluorophores are excited. However, when the vesicle is transformed into an autolysosome, the GFP emission decreases due to the acidity of this compartment [62]. In this assay, the proportion (as a percentage) of simple (red) or double-stained (yellow) structures relative to the total number of vesicles allows the determination of any alteration in the autophagic flux.

**Figure 5.**
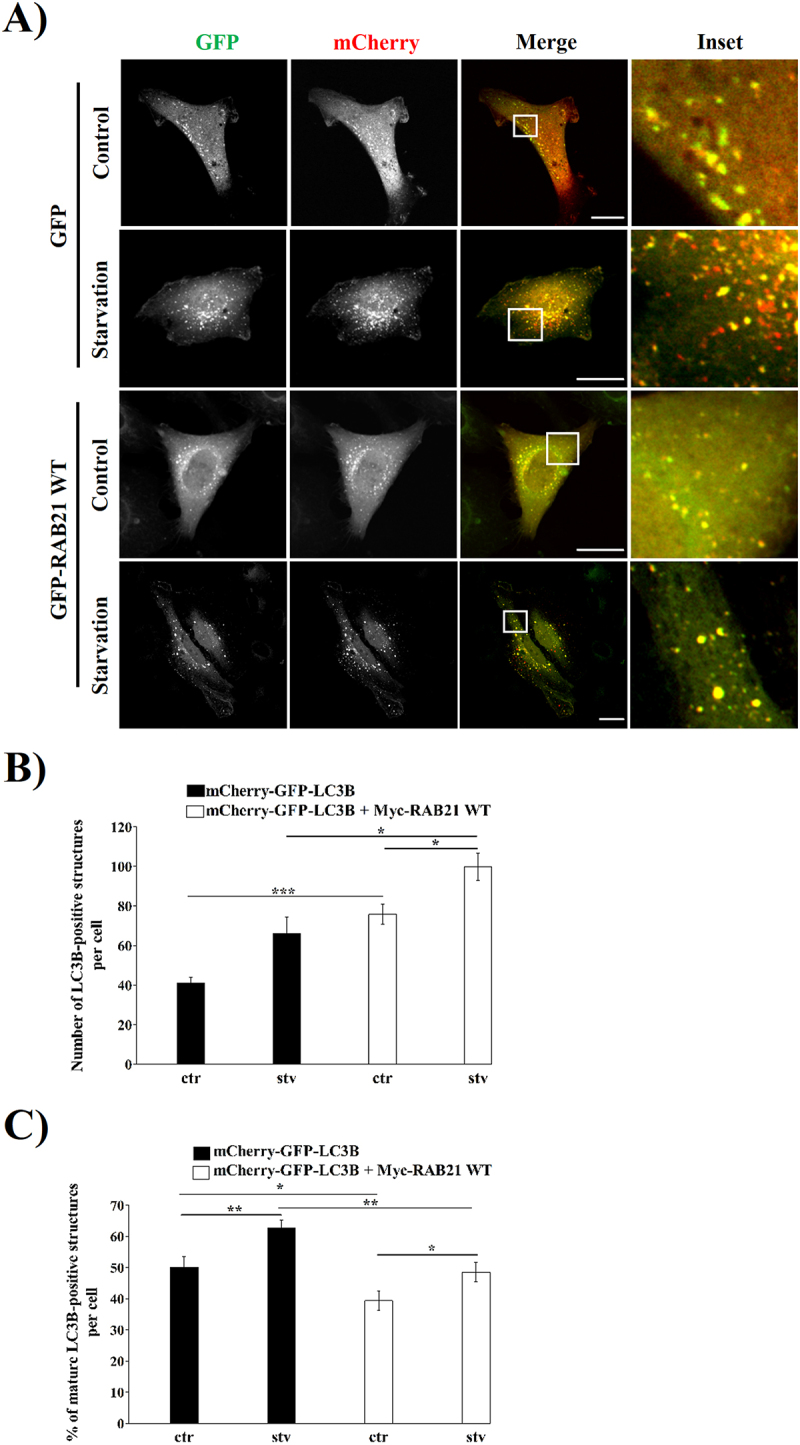
Overexpression of Myc-RAB21 WT promoted the increase of immature structures decorated with LC3 per cell. (A) Representative images of HeLa cells overexpressing a mCherry-GFP-LC3B construct were incubated in control (ctr) or starvation (stv) conditions for 2 hours, co-transfected or not with Myc-RAB21 WT. Scale bar: 30 µm. (B) Quantification of LC3-positive structures at the whole cell. (C) Quantification of the percentage of mature LC3-positive structures per cell. All the graphs show the mean ± SEM of at least three independent experiments. Statistical analyses were done using raw or transformed data and a 2-way ANOVA was performed. In cases where normality was not reached, non-parametric tests were used. Significance: *p ≤ 0.05, **p ≤ 0.01 and ***p ≤ 0.001.

To further demonstrate that the autophagic flux was affected by RAB21 WT overexpression, the mCherry-GFP-LC3B tandem protein was used [62,63]. mCherry-GFP-LC3B was transfected alone or together with Myc-RAB21 WT in HeLa cells and then control or starvation stimulus was applied for 2 hours. As shown in Figure 5(A,B), the total amount of LC3-positive structures (yellow and red dots) was significantly increased when Myc-RAB21 WT was expressed in both control and starvation conditions. Additionally, the percentage of mCherry-positive structures (only red-stained vesicles, i.e. autolysosomes) relative to the total LC3-positive structures was quantified in cells overexpressing mCherry-GFP-LC3B and Myc-RAB21 WT (Figure 5(C)). Of note, a significant decrease of the percentage of mature autophagic structures was observed in Myc-RAB21 WT overexpressing cells in comparison with cells overexpressing only mCherry-GFP-LC3B. Taken together, our results suggest that overexpression of RAB21 WT altered the autophagic flux, producing a marked decrease in the number of mature autophagic structures and favoring a significant increase in the number of immature autophagic vesicles, such as autophagosomes or amphisomes.

### Overexpression of RAB21 WT promotes an increase in DFCP1-positive structures in basal and autophagy-stimulated conditions

On the basis of our observation of an alteration of the autophagic flux in cells overexpressing RAB21^T33N^, whereas no increased level of LC3B-II form was found in the presence of Bafilomycin A1 (Figure 4(A,B)), and given the fact that overexpression of RAB21 WT leads to an accumulation of immature autophagosomes in control or starvation conditions (Figure 5(C)), we decided to study the possible role of RAB21 in the biogenesis of the autophagosomes.

**Figure 6.**
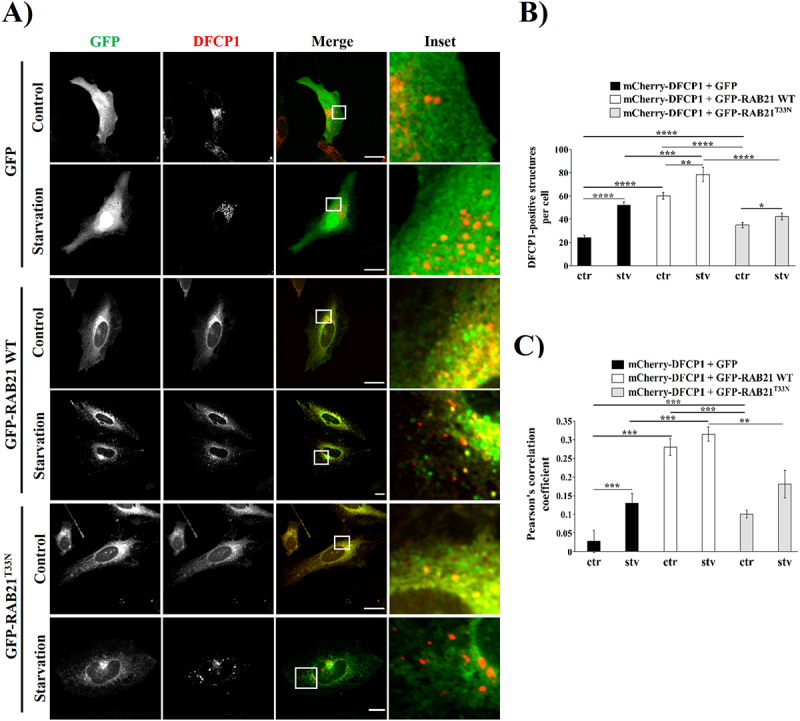
Overexpression of GFP-RAB21 WT promoted the increase of DFCP1-positive structures decorated with LC3. (A) Representative images of HeLa cells overexpressing a mCherry-DFCP1 construct co-transfected with GFP, GFP-RAB21 WT or its constitutively inactive mutant (GFP-RAB21^T33N^), and then incubated in control (ctr) or starvation (stv) conditions for 2 hours. Scale bar: 30 µm. (B) Quantification of the number of DFCP1-positive structures per cell. (C) Colocalization analysis is shown by Pearson’s correlation coefficient at the whole cell. All the graphs show the mean ± SEM of at least three independent experiments. Statistical analyses were done using raw or transformed data prior to a 2-way ANOVA. In cases where normality was not reached, non-parametric tests were used. Significance: *p ≤ 0.05, **p ≤ 0.01, ***p ≤ 0.001 and ****p ≤ 0.0001.

As previously reported in the literature, LC3-positive isolation membranes were found to emerge from a DFCP1-positive, ER-associated compartment called the omegasome [1,2,16,19]. To address the possible role of RAB21 at the early steps of autophagosome formation, HeLa cells overexpressing DFCP1 and RAB21 WT or its constitutively negative mutant were submitted to control or starvation conditions. HeLa cells overexpressing GFP-RAB21 WT significantly display an increased number of mCherry-DFCP1-positive structures in comparison with cells overexpressing the GFP or the GFP-RAB21^T33N^ constructs in both control and starvation conditions (Figure 6(A,B)). Overexpression of CFP-DFCP1 and Myc-RAB21 WT also showed the same tendency (Fig. S6). This result shows that the overexpression of RAB21 WT induces a marked increase in DFCP1 structures in both control and starvation conditions, which is impaired by the overexpression of the inactive form of RAB21. Interestingly, colocalization analysis demonstrated that overexpressed GFP-RAB21 WT significantly colocalized with mCherry-DFCP1-positive structures in both control and starvation conditions in comparison to the constitutively inactive mutant (Figure 6(C)). Moreover, there were no significant differences between the amounts of RAB21-positive structures per cell (Fig. S6C).

**Figure 7.**
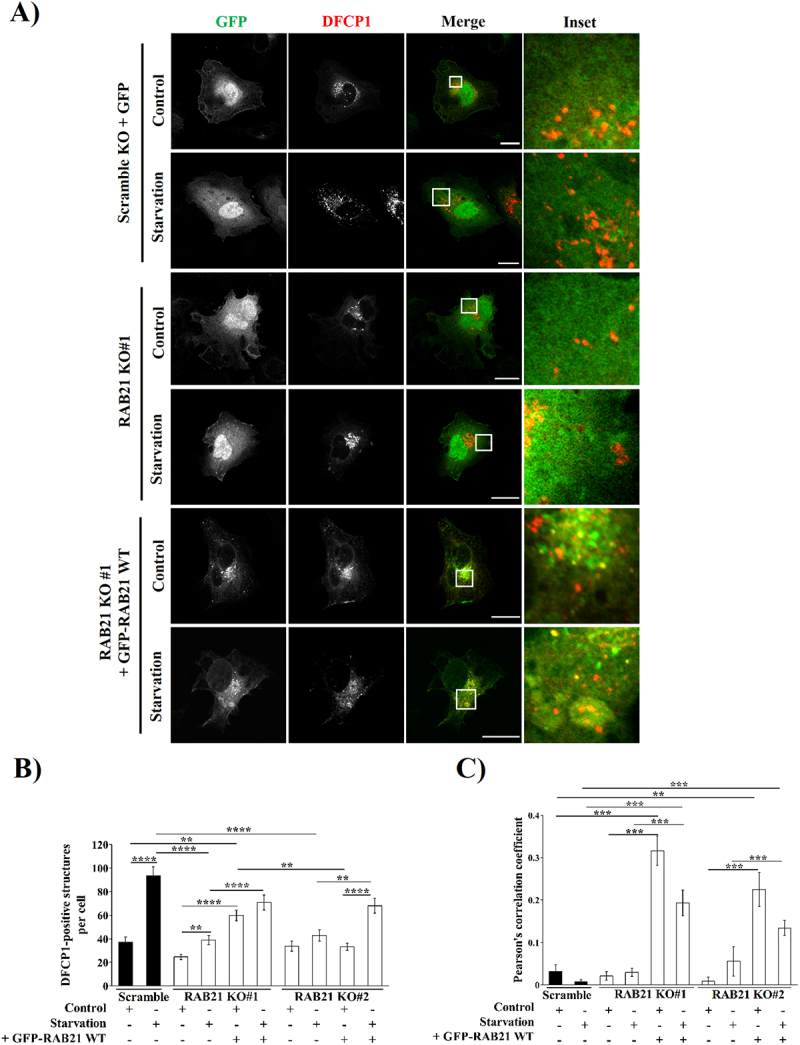
RAB21 KO cells abolishes the increase of DFCP1-positive structures decorated with LC3 after starvation. (A) Representative images of scramble or RAB21 KO#1 heLa cells overexpressing a mCherry-DFCP1 construct co-transfected with GFP or GFP-RAB21 WT, and then incubated in control or starvation conditions for 2 hours. Similar images were obtained with RAB21KO#2 clones (data not shown). Scale bar: 30 µm. (B) Quantification of the number of DFCP1-positive structures per cell. (C) Colocalization analysis is shown by Pearson’s correlation coefficient at the whole cell. All the graphs show the mean ± SEM of at least three independent experiments. Statistical analyses were done using raw or transformed data prior to a 2-way ANOVA. In cases where normality was not reached, non-parametric tests were used. Significance: *p ≤ 0.05, **p ≤ 0.01, ***p ≤ 0.001 and ****p ≤ 0.0001.

To further demonstrate the importance of an active RAB21 protein in autophagosome biogenesis, Scramble KO or RAB21 KO HeLa cells were transfected with mCherry-DFCP1 and GFP-empty vector or GFP-RAB21 WT and submitted to control or starvation conditions for 2 hours (Figure 7(A)). Microscopy analyses demonstrated that RAB21 KO cells had a significant decrease in the number of DFCP1-positive structures per cell after starvation stimulus, a phenotype that was completely rescued with the overexpression of RAB21 WT (Figure 7(B)). As expected, the complementation of the RAB21 KO cells led to a recovery in the colocalization between GFP-RAB21 WT and mCherry-DFCP1 (Figure 7(C)), reaching similar levels to the overexpression experiment (Figure 6(C) vs. Figure 7(C)) as well as the number of RAB21-positive structures per cell (Fig. S6D).

Altogether, these results suggest that RAB21 is likely required for the biogenesis of autophagosomes under both control and starvation conditions.

### RAB21 GEF, VARP, colocalizes with LC3 after autophagy stimulation

As mentioned above, VARP is the GEF of RAB21, involved in melanogenesis and VAMP7 regulation in a spatiotemporal manner [64]. Thus far, the only association of VARP with autophagy in mammalian cells comes from a study that revealed that VARP knockdown cells showed a decrease in LC3-positive dots at basal conditions as well as the total LC3B-II protein amount upon Bafilomycin A1 treatment [32].

**Figure 8.**
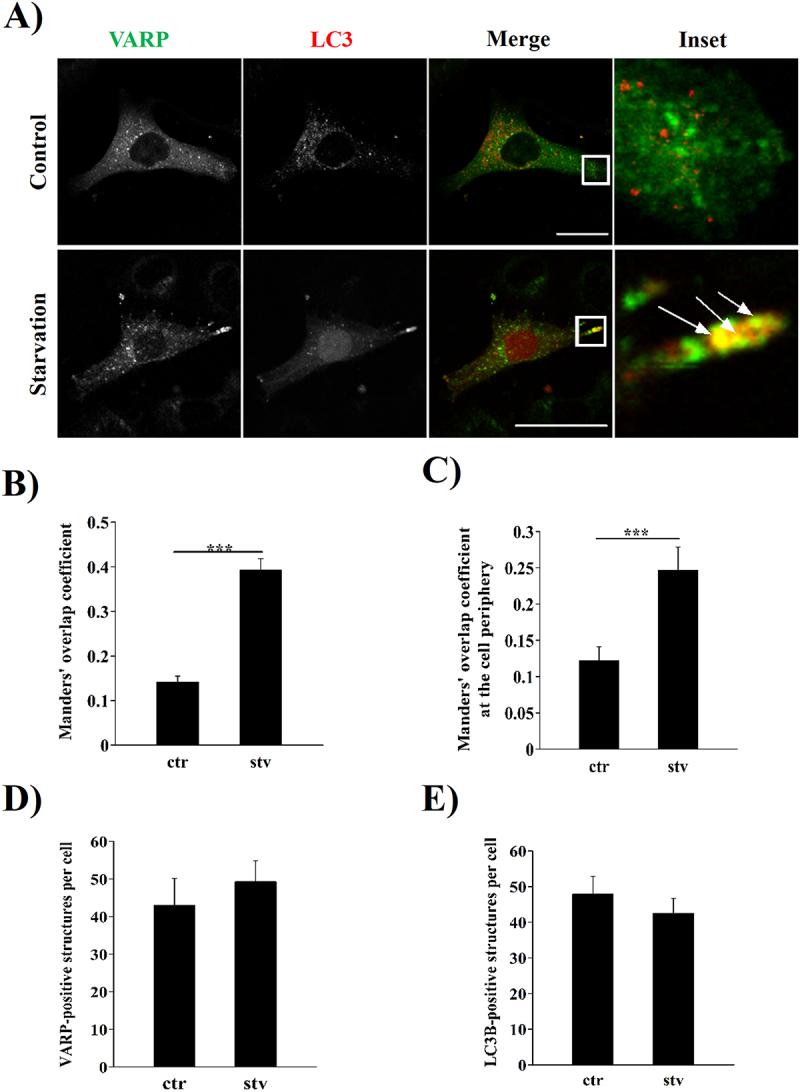
GFP-VARP and RFP-LC3 colocalized after starvation stimulus in HeLa cells. (A) Representative images of HeLa cells overexpressing a GFP-VARP construct co-transfected with RFP-LC3 and incubated in control (ctr) or starvation (stv) conditions for 2 hours. Arrows show colocalization between LC3 and VARP at the cell periphery. Scale bar: 30 µm. Colocalization analysis is shown by Manders’ overlap coefficient at the whole cell (B) or at the cell periphery (C). The number of VARP or LC3-positive structures at the whole cell or at the cell periphery is shown in (D) and (E), respectively. All the graphs show the mean ± SEM of at least three independent experiments. No significant differences were found. Statistical analyses were done using raw or transformed data prior to a T-Test. In cases where normality was not reached, non-parametric tests were used. Significance: ***p ≤ 0.001.

Because our results lead to a possible role of RAB21 in autophagy, we decided to study the potential role of VARP in the trafficking of autophagic structures. HeLa cells overexpressing GFP-VARP and RFP-LC3 were incubated in a control or starvation medium for 2 hours. As shown in Figure 8(A,B) a significant increase in the colocalization between these proteins was observed in starved cells. Similar results were obtained when the colocalization analysis was performed at the cell periphery under the same conditions mentioned above (Figure 8(C)). Notably, the total amount of VARP or LC3 positive structures per cell in control or starvation conditions showed no significant differences (Figure 8(D,E)). These results suggest that starvation induced an increased number of VARP-LC3-positive vesicles, both at whole cell level and at the cell periphery.

### Starvation increases the colocalization of RAB21 and VARP

**Figure 9.**
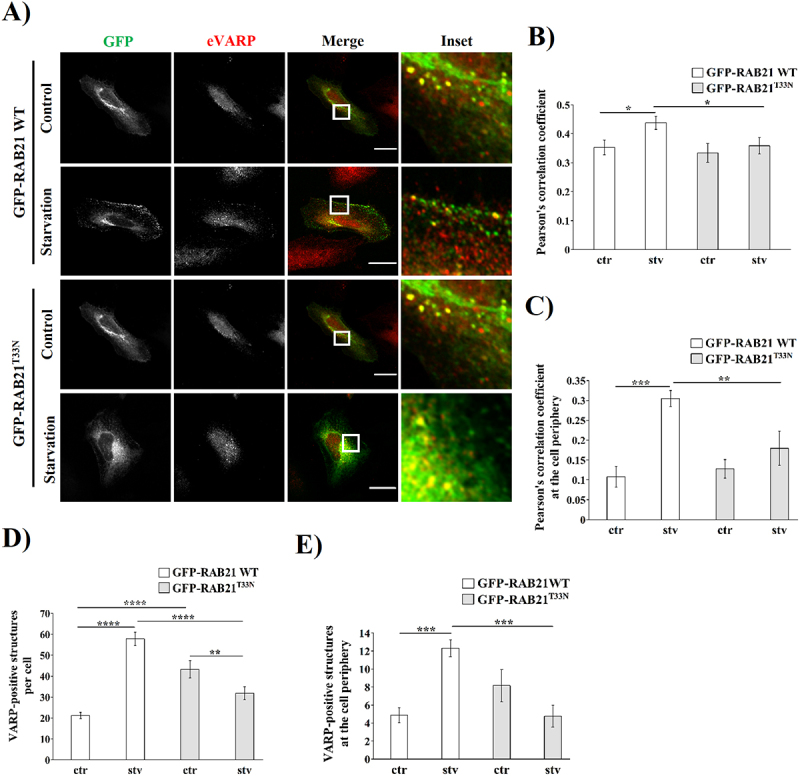
Redistribution to cell periphery and increased colocalization after starvation is impaired in RAB21^T33N^ overexpressing HeLa cells. (A) Representative images of HeLa cells overexpressing a GFP-RAB21 WT or its constitutively inactive mutant RAB21^T33N^ construct and incubated in control (ctr) or starvation (stv) conditions for 2 hours. Endogenous VARP (eVARP) was detected by immunostaining. Scale bar: 30 µm. Colocalization analysis is shown by Pearson’s correlation coefficient at the whole cell (B) or at the cell periphery (C). The number of eVARP-positive structures at the whole cell or at the cell periphery is shown in (D) and (E), respectively. All the graphs show the mean ± SEM of at least three independent experiments. Statistical analyses were done using raw or transformed data prior to a 2-way ANOVA. Significance: *p ≤ 0.05, **p ≤ 0.01, ***p ≤ 0.001 and **** *p* ≤ 0.0001.

Because our results point to a role of RAB21 and its GEF VARP in autophagy, we decided to evaluate if the overexpression of the RAB21 constitutively inactive mutant affects the location of VARP-positive structures. To address this experiment, HeLa cells overexpressing GFP-RAB21 WT or GFP-RAB21^T33N^ were incubated in control or starvation conditions for 2 hours and then the endogenous VARP (eVARP) was detected by indirect immunofluorescence. Overexpression of GFP-RAB21 WT led to a significant colocalization increase with eVARP upon starvation in comparison with the control condition as well as the overexpression of the constitutively inactive mutant of RAB21 (Figure 9(A,B)). Notably, greater differences were observed when colocalization analysis was performed at the cell periphery (Figure 9(C)). In addition, overexpression of GFP-RAB21^T33N^ significantly decreased the number of VARP-positive structures per cell as well as at the cell periphery in starved cells (Figure 9(D,E)). These results suggest that RAB21 and VARP participate in autophagy, and an active RAB21 is necessary to redistribute VARP-positive structures after starvation.

## Discussion

Here we took advantage of KO of VAMP7, VARP and RAB21 cells as well as overexpression of RAB21 WT or its constitutively inactive mutant to propose a function of RAB21 in autophagy. In support of this, we found that RAB21 KO impaired the amphisome secretion of ATP, the generation of LC3B-II, and the formation of LC3- and DFCP1-positive structures. In addition, RAB21 WT overexpression rescued the phenotype of RAB21 KO, and expression of the constitutively inactive mutant of RAB21 phenocopied RAB21 KO phenotype. Starvation further increased RAB21-VARP colocalization. Altogether, these results confirm the role of the VAMP7-VARP-RAB21 molecular network in ATP-amphisome secretion and further allow us to propose an additional role of RAB21 in the early steps of autophagy.

RAB21 has been previously involved in endosomal transport and cell proliferation, adhesion, and invasion [29,32,35–37,57,65–69]. Pellinen *et al*. (2006) also found that RAB21 decorates a small percentage of double-membrane structures in MDA-MB231 cells [35]. In agreement, our results here showed that a population of RAB21-positive structures colocalized with LC3 both in basal and autophagy-stimulating conditions. Additionally, this colocalization appeared to be augmented at the cell periphery after starvation with RAB21 WT but not with RAB21^T33N^ overexpressing cells (Figure 2(B,C)). Our results showed that RAB21 is likely implicated in the redistribution of autophagic vesicles from the perinuclear region to the cell periphery and must be activable in order to play this function, because overexpression of RAB21^T33N^ significantly diminished the colocalization between RAB21 and LC3 at the cell periphery (Figure 2(C)). A recently published article showed that GFP-RAB21 stably expressing HeLa cells had no noticeable colocalization with LC3 in control conditions [31]. This apparent discrepancy might only be related to slight differences in experimental conditions related to stable vs. transient transfection. As discussed below, Pei et al. (2023) also concluded on a role of RAB21 in autophagy, proposing a mechanism involving retromer-mediated recycling of SLC2A1/GLUT1, already known to involve VARP [34]. Indeed, Jean et al. (2015) proposed that RAB21 regulates the lysosome-autophagosome fusion coupled with its GEF MTMR13 and the v-SNARE VAMP8 after starvation. They found that RAB21 depletion promotes LC3B-II accumulation in non-acidic vesicles, similar to Bafilomycin A1 treatment in basal conditions [32]. Nevertheless, they did not evaluate the autophagic flux after starvation stimulation. Accordingly, RAB21 KO HeLa cells were found to have an increased number of LC3-positive structures in comparison with WT cells both control as well as amino acid starvation [31]. Our results showed that RAB21^T33N^ as well as RAB21 KO HeLa cells diminished the number of LC3-positive structures and LC3B-II concentration after starvation (Figures 2(D) and 3(D–G)). Moreover, our results showed that the autophagic flux was altered by the overexpression of RAB21^T33N^ at basal conditions (Figure 4(A,B)). Moreover, we found that overexpression of RAB21^T33N^ diminished the total amount of LC3B-II at basal conditions. This decrease may be associated with an active RAB21 function in the early steps of autophagy, in addition to its role in lysosome-autophagosome fusion. Conversely, Pei et al. (2023) showed that RAB21 KO cells as well as HeLa cells overexpressing GFP-RAB21^T33N^ had an altered autophagy flux in which an increase in LC3B-II levels was observed in amino acid starvation. This discrepancy could be explained by the use of different full and starvation media. As mentioned in Materials and Methods in the present article, the starvation medium used consists briefly of buffered saline solution (EBSS) without any addition of serum, glucose, or amino acids, whereas in Pei et al. (2023) used amino acids depletion only. Further analyses of the different conditions of autophagy induction are required to fully understand the potential differences. Nevertheless, both Pei et al. (2023) and the present study agree on the role of RAB21 in autophagy early steps.

To further elucidate the role of RAB21 in the autophagy flux, we decided to investigate the acidity of RAB21 and LC3-positive vesicles by two different approaches. First, the DQ Red BSA internalization and degradation experiment demonstrated that the overexpression of GFP-RAB21 WT did not increase the acidic compartments in the cell (Figure 4(C,D)). Second, we used a mCherry-GFP-LC3B tandem protein, a tool widely used to assess the acidity of LC3-positive structures [62]. Co-expression of Myc-RAB21 WT and mCherry-GFP-LC3B constructs resulted in a significant accumulation of nonacidic LC3-positive vesicles at basal and starvation conditions in comparison to GFP transfected cells (Figure 5), in agreement with previous work showing that RAB21 KO cells led to an increase in acidic compartments [31]. Our results regarding mCherry-GFP-LC3 overexpression and DQ Red BSA experiment are consistent with the hypothesis that RAB21 could lead to an increase in immature autophagosomes (or amphisomes) rather than autolysosomes in HeLa cells, suggesting a possible role for RAB21 in early steps of autophagosome biogenesis.

In an attempt to elucidate the implication of RAB21 in autophagosome formation, we overexpressed GFP-RAB21 and mCherry-DFCP1, which is involved in autophagosome membrane closure [1,2,19]. We found that overexpressed GFP-RAB21 WT but not the constitutively inactive mutant nor the RAB21 KO cells significantly increased the DFCP1-positive structures at basal and starvation conditions (Figure 6(A,B)), in agreement with our autophagic flux results. In addition, the overexpression of RAB21 WT in RAB21 KO cells led to a recovery of DFCP1 puncta (Figure 7(A,B)). Moreover, a pull-down assay followed by mass spectrometry in HEK293 cells revealed that RAB21 might directly interact with ATG7, an E3-like ligase involved in LC3 processing and lipidation [70], supporting the hypothesis that RAB21 might be implicated in the first steps of autophagy. We consider that RAB21's regulatory proteins could be differentially involved in autophagosome biogenesis and/or its fusion with the lysosome, potentially giving the spatio-temporal specificity required for the action of RAB21. It is important to highlight that our study supports the involvement of RAB21 at the level of autophagosome biogenesis, and more work is needed to reveal the exact implication of RAB21, particularly its effectors and interacting partners.

RAB21 has been extensively studied in relation to endocytosis. Nevertheless, RAB21 has also been described in VAMP7-positive neutral vesicle transport from the cell center to the cell periphery, where it fuses with the plasma membrane in neurites [29,57]. Importantly, our group has previously defined that MultiVesicular Bodies (MVBs) that fuse with autophagosomes (forming a new membrane compartment termed amphisomes) are delivered to the cell periphery in a KIF5A and autophagy induction-dependent manner [22]. Additionally, KIF5A interacts with VARP and mediates the transport of VAMP7 to the cell periphery [30]. The above works support the idea that RAB21 redistribution to the cell periphery is more likely due to exocytosis than endocytosis after autophagy stimulation. We cannot exclude the hypothesis that both events can occur simultaneously, and we consider that more work is needed regarding this putative new role of RAB21 in the exocytosis of autophagy-related vesicles. Our live imaging results point to an increase in exocytosis rather than in endocytosis trafficking in RAB21 WT overexpressing cells after starvation (Fig. S4 and Movies 01 and 02). Given the fact that we found a redistribution of RAB21-LC3-positive vesicles to the cell periphery in starved cells, we conducted an exocytosis assay using ATP as cargo, as we previously demonstrated that is released after autophagy induction [22]. Our results demonstrate that RAB21 is involved in ATP exocytosis because RAB21^T33N^ overexpression or RAB21 KO cells significantly decreased the total amount of eATP (Figure 1(C,D) and Fig. S1). This is in good agreement with the role of RAB21 and VARP in the transport of VAMP7 to the cell periphery [23,29,30].

VARP has been studied in relation to melanogenesis, regulating several steps of vesicle trafficking for and from the melanosome [33,71,72]. In addition, Jean *et al*. (2015) have investigated how VARP knockdown could affect autophagy. VARP silencing was reported to promote a decrease in LC3-positive vesicles without any change after Bafilomycin A1 treatment [32]. In agreement, our results showed that, after starvation, HeLa cells overexpressing GFP-VARP WT and RFP-LC3 significantly increased their colocalization both at the whole-cell level as well as at the cell periphery (Figure 8). Because VARP is tightly related to RAB21 and VAMP7, we hypothesize that VARP could also be implicated somehow in the biogenesis of autophagosomes, and more studies are needed in order to confirm this hypothesis. Because VARP is one of the RAB21 GEFs, an overt colocalization at the whole cell level is expected at basal conditions [29,30,33,34,71,72]. Given the fact that VARP and RAB21 separately colocalized at some extent with LC3 (as shown in Figures 2 and 8), we wondered if there is any change in colocalization between RAB21 and VARP after autophagy stimulation. Of note, the overexpression of GFP-RAB21 WT led to a significant increase in its colocalization with endogenous VARP in starved HeLa cells, whereas the overexpression of RAB21^T33N^ showed no significant increase both at whole cell as in the cell periphery (Figure 9(B,C)), suggesting that an active form of RAB21 is needed. Interestingly, the constitutively inactive mutant of RAB21 led to a significant decrease in the number of VARP-positive vesicles both at cell periphery as well as the whole cell level (Figure 9(D,E)). One possible explanation is that RAB21^T33N^ is sequestering VARP at the perinuclear region [28,29], highlighting the importance of an active form of RAB21 in starvation-induced processes. Nevertheless, further studies are needed to fully understand the involvement of VARP and RAB21 in autophagosome biogenesis.

As mentioned earlier, VARP has been included in a model in which it interacts with RAB21, VAMP7, and KIF5A to transport vesicles from the perinuclear zone to the cell periphery in neuronal cells [30]. Additionally, VARP is important for the recycling of Rab32/38 and VAMP7 from the melanosome membrane [64]. Since our previous work positioned VAMP7 and KIF5A in the transport of ATP-charged vesicles, we studied the implication of VARP in ATP exocytosis. We found that VARP KO slightly diminished the eATP concentration after starvation in comparison with Scramble KO, evidencing its possible role in autophagic exocytosis of ATP (Figure 1(B) and Fig. S1E). Of note, the overexpression of GFP-RAB21 WT in VARP KO cells did not compensate for the lack of VARP (Figure 1(B) and Fig. S1E). VAMP7, another component of the machinery implicated in ATP-loaded vesicles transport to the plasma membrane, has also been described as an important player in the ATP autophagy secretion [22]. The depletion of VAMP7 impaired the increase in ATP release after starvation (Figure 1(A) and Fig. S1D), in agreement with our previous results in which quinacrine-positive vesicles (an ATP probe) were transported to the cell periphery where they fused with the plasma membrane [22]. Additionally, VAMP7 KO cells overexpressing GFP-RAB21 WT did not recover the secretory phenotype (Figure 1(A) and Figure S1D), supporting the role of VAMP7 as partner of RAB21 and VARP in ATP autophagy-dependent secretion. Moreover, KIF5A has also shown its possible implication in ATP-autophagic exocytosis, since the overexpression of the ID slightly decreased the eATP after starvation, reaching almost basal levels (Figure S2). This is in accordance with previous results, showing that KIF5A is the motor protein implicated in the transport of VAMP7-positive vesicles from the cell center to the cell periphery [22,23,30].

Extracellular ATP has been implicated in a variety of cellular processes, from Immunogenic Cell Death (ICD) to differentiation [51,52,73]. We found basal values of eATP of approximately 0.2 nM, which increased up to 0.325 nM after 2 hours of starvation induction (Figure S1). These values are consistent with the eATP found in previous works in several cell lines [52,73–75]. Thus, starvation is capable of inducing the secretion of ATP, but it is still unknown which is the fate of this nucleotide in the context of *in vitro* experiments. Here, we demonstrated that RAB21, VARP, VAMP7 and KIF5A are involved in the exocytosis of ATP, establishing a possible model in which these proteins could promote non-conventional ATP secretion after induction of autophagy by starvation. These findings open a wide, unknown, and interesting field that has been poorly investigated thus far.

Finally, this article gives new insights regarding the importance of traffic regulators in autophagy and ATP exocytosis. Future investigations should study deeply the importance of RAB21 and VARP in autophagy early steps and in unconventional exocytosis as well.

## Material and methods

### Reagents

Ammonium chloride (NH4Cl; Dalton 0,016,900,250), Ammonium Persulfate (Bio-rad 1,610,700), ATP (Sigma, A2383-1 G), Bovine Serum Albumin (BSA; Biowest, A0296), Calcium Chloride (Anedra 10,043-52-4), DQ™ Red BSA (Thermo Fisher Scientific, D12051), Dulbecco’s Modified Eagle Medium (DMEM; Thermo Fisher Scientific 12,100,046), Enhanced Chemiluminescence Kit (ECL; Merck, WBKLS0100), Ethylenediaminetetraacetic acid (EDTA; Promega, H5032), Fetal Bovine Serum (FBS; Natocor), Glucose (Anedra, 50-99-7), Luciferase-Luciferin kit assay (Molecular Probes 16,176), Magnesium Sulfate (Tetrahedron), Mowiol (Calbiochem 475,904-100 GM), Ni-NTA agarose 6×His-tagged purification kit (Qiagen 30,210), Nitrocellulose membrane (GVS 1,213,314), Paraformaldehyde (Cicarelli 1,088,211), Penicilin/Streptomycin (Thermo Fisher Scientific 15,140,122), Potassium Chloride (Biopack, 7447-40-7), Potassium Phosphate Diacid (Tetrahedron), Saponin (Dalton), Sodium Bicarbonate (Anedra, 144-55-8), Sodium Chloride (Anedra, 7647-14-5), Sodium Chloride (NaCl; Anedra, 7169), Sodium Phosphate Dibasic (Biopack, 7558-79-4), Tris-Chloride buffer (Promega, H5121), Triton 100× (Biopack, 3402.13.00), Trypsin (Thermo Fisher Scientific 25,200,056).

### Plasmids

GFP-Vector and RFP-LC3 were generated as indicated in our previous publications [22,25]. GFP-VARP WT, GFP-RAB21 WT, Myc-RAB21 WT and GFP-RAB21^T33N^ were previously reported [29,30]. 6×His-GBP was kindly donated by Christian Vannier (Institute of Psychiatry and Neuroscience of Paris, Paris, France). mCherry-GFP-LC3B was kindly provided by Dr. Terje Johansen (University of Tromsø, Norway), CFP-DFCP1 and mCherry-DFCP1 were generous gifts from Dr. Tamotsu Yoshimori (Osaka University, Japan).

### Cell culture and transfection

HeLa cells were maintained according to ATCC’s protocols. Briefly, HeLa cells were grown in DMEM supplemented with 10% FBS (full nutrient medium), streptomycin (50 μg/mL), and penicillin (50 U/mL) at 37°C under humidity and 5% CO2 atmospheric conditions. For some experiments, cells were incubated in EBSS’s starvation medium (0.002 M Calcium Chloride, 0,003 M Magnesium Sulfate, 0.005 M Potassium Chloride, 0.117 M Sodium Chloride, 0,008 M Sodium Dibasic Phosphate, 0,0055 M Glucose and 0,026 M Sodium Bicarbonate, pH = 7,4) for 2 hours or in full nutrient medium as a basal condition for 4 hours. In addition, HeLa cells were transiently transfected with the plasmids mentioned above using Lipofectamine 2000 (Thermo Fisher Scientific 11,668,027) or by nucleoporation with an Amaxa Nucleofector II (IHEM, Mendoza, Argentina), as indicated by the manufacturer. Importantly, the cells were incubated in the different treatments 24 hours post transfection, after confirming a high and efficient transfection.

### Autophagic flux experiment

Autophagic flux was evaluated using 100 nM Bafilomycin A1 (Sigma-Aldrich, SML1661) added to the stimuli and culture conditions described above.

### DQ red BSA experiment

The ability of HeLa cells to endocytose and degrade the self-quenched red Bodipy dye conjugated to BSA (DQ Red BSA) was used to measure lysosome function. DQ Red BSA requires enzymatic cleavage in an acidic intracellular compartment to generate a highly fluorescent product, which can be monitored by confocal microscopy. Transfected HeLa cells plated on proper coverslips were incubated for 2 hours at 37 °C with DQ Red BSA (10 μg/mL in DMEM containing 10% FBS) to ensure that the reagent reached the lysosomal compartment. Cells were then washed twice with PBS (137 mm Sodium Chloride, 1.8 mm Potassium Phosphate Diacid, 2.7 mm Potassium Chloride and 10 mm Sodium Phosphate Dibasic, pH = 7,4), and incubated under different experimental conditions.

### Indirect immunofluorescence

HeLa cells plated on proper coverslips were fixed with 0.2 mL of 4% paraformaldehyde (dissolved in PBS) for at least 20 min at room temperature. Then, the coverslips were washed with PBS and quenched with 50 mm NH_4_Cl in PBS for 20 min. Next, cells were permeabilized with a 1% saponin solution also containing 1% BSA (blocking buffer). After BSA blocking, cells were incubated with the primary antibodies for 1 hour or overnight. Then, cells were washed three times in a blocking buffer and subsequently incubated for at least 1 hour with the fluorophore-conjugated secondary antibody. Overexpressed Myc-tag was detected using antibodies against Myc (1:200 dilution; Sigma-Aldrich, M4439). Bound antibodies were subsequently detected by incubation with Cy3 goat anti-mouse secondary antibody (dilution 1:500; Abcam, ab97035). Coverslips were finally mounted on glass slides using a Mowiol solution and then examined by fluorescence confocal microscopy.

### Generation of VARP, VAMP7 and RAB21-depleted HeLa cell lines

To produce a HeLa cell line knockout for VARP or VAMP7 by CRISPR/Cas9, we generated RNA-CAS9 guide constructs based on the published protocol [76]. Briefly, oligonucleotides targeting sequences near the translation starting site of the gene of interest (ANKRD27 or VAMP7) were designed using the “RGEN Cas Designer” web-based tool [77]. To limit off-targets, sequences of oligo with ≤ 2 putative mismatches throughout the whole genome or an “out of frame” score < 66 were excluded. The primer sequences for each clone are shown in Table S1. Each pair of oligonucleotides (10 mm) was heated at 95°C for 5 min and annealed by ramping down the temperature from 95°C to 25°C at 5°C min-1. Annealed oligonucleotides were ligated into the pSpCas9(BB)-2A-Puro (PX459) vector (Addgene 62,988, Feng Zhang) using the BbsI sites. After validation by sequencing, the targeting constructs were transfected into HeLa cells. At 24 hours post-transfection, cells were diluted, and transfected cells were selected by 1 μg/mL Puromycin (Sigma, P8833) addition. The selected populations were then seeded into a 96-well plate at virtually 1 cell per well. RAB21 KO cells were developed by Tacgene facility (MNHN) using sgRNA from Xinjun Zhang lab [31]. Clones derived from single cells for at least two different clones were obtained and screened for expression deficiency by immunoblotting. RAB21 polyclonal antibody (Abclonal, A12095).

### Fluorescence microscopy and image processing

HeLa cells prepared for imaging were analyzed by confocal microscopy using a FV1000-EVA Olympus Confocal Microscope or a Nikon C1 Confocal Microscope (IHEM, Mendoza, Argentina). Additionally, a 60X objective lens and 2X digital zoom (when necessary) were used for image acquisition. Then, 1024 × 1024or 500 × 500pixel RGB images were processed using ImageJ or FIJI free software for particle analysis and for colocalization analyses. Briefly, the puncta quantification was made using the following protocol: First, RGB images were transformed to an 8-bit image, and color-channels were separated. Then, Brightness and Contrast were manually adjusted using the HiLo LUT. After this, the image was duplicated and a manual threshold was applied, followed by the selection of the specific ROI (whole cell without nucleus or periphery ROI). Finally, the particle analysis of ImageJ software was used to automatically obtain the amount of LC3-positive structures inside the ROI. For the colocalization analysis, a Manders and Pearson’s coefficients-based plugin was used. The same image processing mentioned above was used, with the addition of a mask creation using the specific ROI prior to the Manders and Pearson’s coefficients-based plugin use.

The region of interest (ROI) for each cell was delimited before the colocalization or particle analysis. Whole-cell ROIs were made by subtracting the nucleus, while periphery ROIs were made using a 10-pixel-wide Selection Brush Tool (Figure S1).

### Live imaging and data processing

HeLa cells were seeded on 20 mm glass coverslips in a 6-well plate. At 80% confluency, the cells were transfected with GFP-RAB21 WT or GFP-RAB21^T33N^ plasmid. Twenty-four hours post-transfection, cells were washed three times with PBS and then they were incubated in full or starvation medium for 2 hours at 37°C. Subsequently, the coverslips were mounted on a time-lapse chamber containing 1 mL of either full or starvation medium and maintained at 37°C until video microscopy was performed. Time-lapse images were acquired every 15 seconds for 10 minutes, using an Olympus FV1000 confocal microscope. The resulting time-lapse stacks were analyzed with ImageJ software. Individual vesicles were manually tracked using the MTrackJ plugin, with a minimum tracked distance of 1.5 microns. Then, a classification of single-vesicle movement was done, in which white tracks represent vesicles moving from the perinuclear region to the cell periphery, while red tracks indicate vesicles moving from the cell periphery to the perinuclear region as well as motionless vesicles. A quantification of white and red track per cell was made and the proportion (%) of total vesicles that display any of the mentioned movements is shown on a stacked graph.

### GFP-Trap

The GFP-Trap assay was performed following the manufacturer’s protocol with some modifications (Chromotek, gtak-20). Basically, after HeLa cells were incubated under autophagic conditions, the cells were harvested and centrifuged at 1000 g for 10 minutes. Next, the pellet was incubated in a lysis buffer for 30 minutes on ice. The solution was subsequently centrifuged, and the pellet was then incubated with the GBP protein between 2 hours and overnight. Next, the solution was incubated with Ni-NTA agarose beads for 30 minutes on ice. After that, the solution was centrifuged and resuspended three times in a washing buffer from the Ni-NTA agarose purification kit. Finally, the pellet was resuspended in a 2X sample buffer and heated in a sand bath for 5 minutes. Samples were taken from input, washing, and binding steps for immunoblotting.

### SDS-PAGE and Western blot

Cells transfected with the plasmids of interest were plated in a 6-multiwell plate and cultured for 48 to 72 hours. Next, cells were washed with a PBS buffer and mechanically detached. Subsequently, cells were centrifuged, and the pellet was resuspended in a Lysis Buffer (Triton 100×, 150 mm NaCl, EDTA, and 1 M Tris-Chloride, pH = 7.5). Then, the samples were stored at −20°C until electrophoresis in polyacrylamide gel was performed. Afterward, the samples were incubated in the sand bath at 95°C for 5 minutes with 4× Sample Buffer. The samples were run using a 15% polyacrylamide gel and then transferred to a nitrocellulose membrane (Biorad System 1,620,117). The membranes were blocked using 5% nonfat milk, PBS, or TBS and a 1% Tween 20 solution for at least 1.30 hour. Primary antibodies were incubated for at least 2 hours. Anti-LC3B (1:1000; Sigma-Aldrich, L7543), Anti-GFP (1:1000; Sigma-Aldrich, AB10145), Rab21 polyclonal antibody (1:1000, Abclonal, A12095), Anti-VARP (1:2000, TG40a from T. Galli’s lab), Anti-GAPDH (1:30000, Sigma, G4595), Anti-VAMP7 (1:1000, SYSY 232,011) and Anti-actin (1:500; Sigma-Aldrich, A2066) were used in this paper. Secondary antibodies (1:10000; Anti-Mouse or Anti-Rabbit HRP; Jackson Lab, 115-035-044 and 111-035-003) were incubated for 1 hour at room temperature, and then the corresponding bands were detected using an Enhanced Chemiluminescence Kit and a chemiluminescence detector (Las-4000 ImageQuant, GE Healthcare, IHEM, Mendoza, Argentina). The imageJ and FIJI free softwares were used to analyze the images obtained by the Gel Analysis plugin.

### Measurement of ATP release

Released ATP in the samples was measured by a Luciferase-Luciferin-based assay, following the manufacturer’s instructions. Briefly, HeLa cells were grown to confluence on p6 multiwell plates. After washing with PBS, cells were incubated at 37°C in full or starvation medium for 2 hours. Supernatant aliquots of 50 μL were collected before and after the treatment incubation and frozen at −20°C. Next, the samples were incubated with 50 μL of firefly luciferin-luciferase in a black 96-well plate. The ATP-dependent chemiluminescent activity was measured in constant darkness using a luminometer called Fluorocount Ascent (Thermo, IHEM, Mendoza, Argentina) or a Spectramax Microplate Reader (Molecular Devices, Paris, France) at 555 nm wavelength. The former one gives Relative Light Units (R.L.U.), while the last one gives the Absorbance of each sample in arbitrary units (A.U.). Each calibration curve was made using ATP at final concentrations of 0, 1, 2, 4, 8, 16, 32, 64 and 128 × 10^−10^ M. The data were processed using Excel to obtain the curve function. Therefore, we could stipulate the concentration of extracellular ATP for each sample.

### Statistical analyses

The free software ImageJ was used for image processing and to obtain the Manders or Pearson’s coefficients of each cell. The transformations were used to normalize the data, and the ANOVA and Tuckey test were performed. In cases where normality couldn’t be reached, non-parametric tests were used, followed by a non-parametric Steel-Dwass multi-comparison *post hoc* test. The results are presented as the mean ± SEM of transformed data from at least three independent experiments. The graphs were made using the free software Kyplot 6.0.

## Supplementary Material

Supplemental Material
