# Supplementary material for: A new role of RAB21 and VARP in autophagy and autophagic exocytosis of ATP
Source: Autophagy Rep. 2025 May 11;4(1):2501365. doi: 10.1080/27694127.2025.2501365 (PMC12077462; doi:10.1080/27694127.2025.2501365)
Supplement: Supplemental Material [file KAUO_A_2501365_SM2786.zip › Supplemental figures Barbosa and Reta et al 2025.docx]

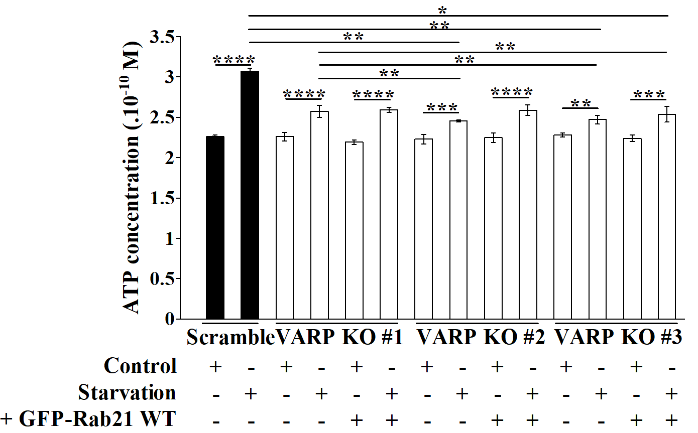

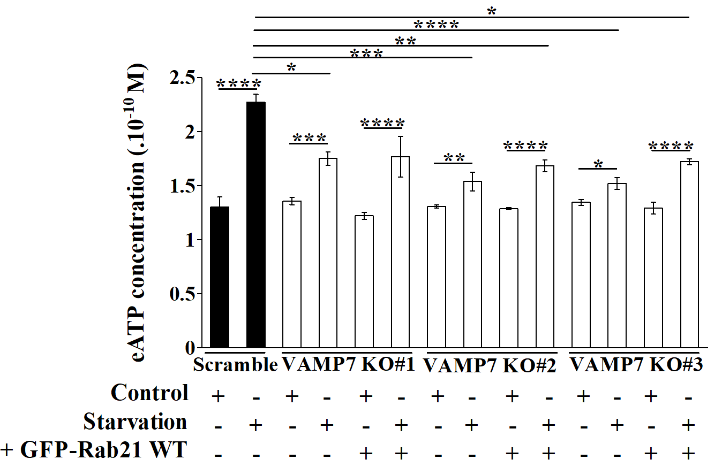

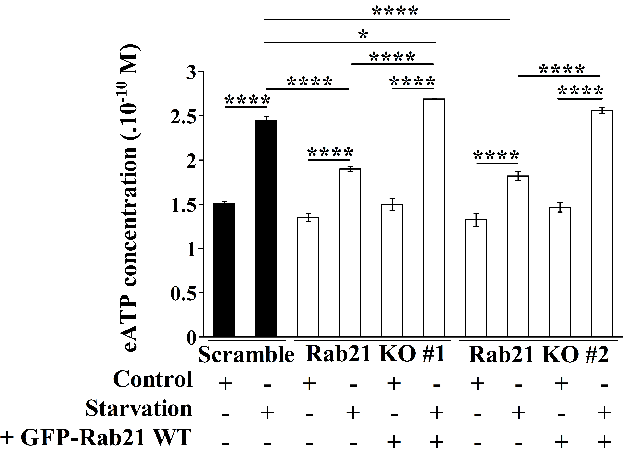

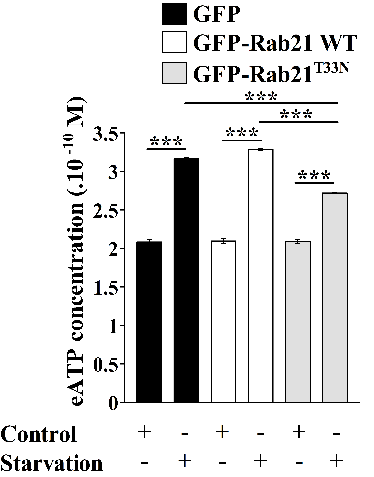


**A)**

**B)**

**F)**

**G)**

**C)**

**I)**

**J)**


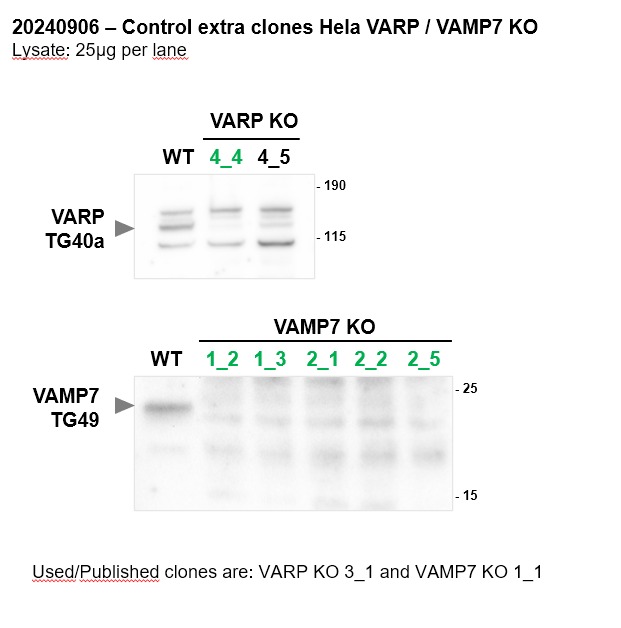


**WT**

**KO**

**#2**

**KO**

**#3**

**VAMP7-**

**TG49**

**-25**

**-15**


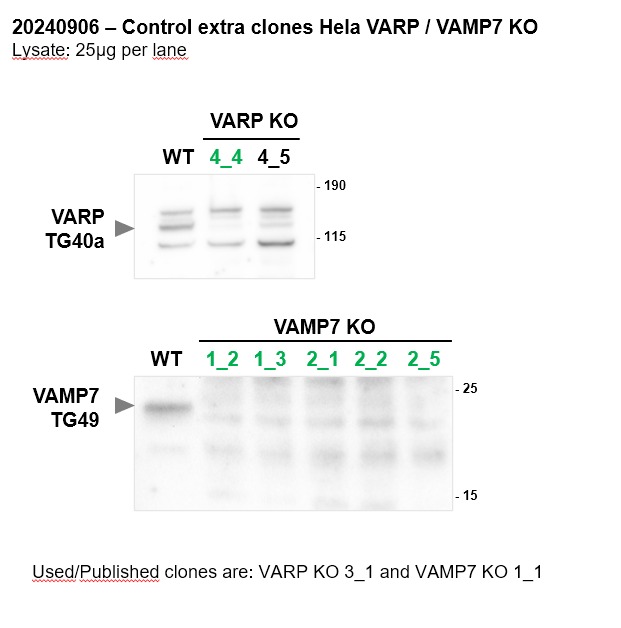


**WT**

**KO**

**#2**

**KO**

**#3**

**VARP-**

**TG40a**

**-190**

**-115**


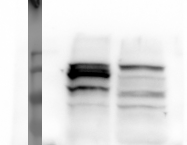

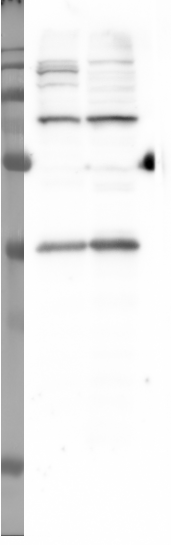


**-130**

**-100**

**-35**

**WT**

**KO**

**#1**

**VARP-**

**TG40a**

**GAPDH-**

**E)**

**D)**


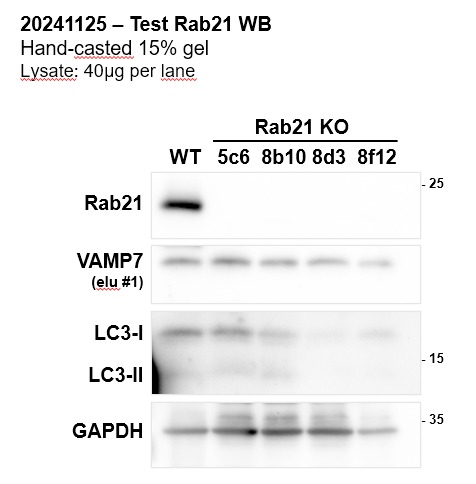

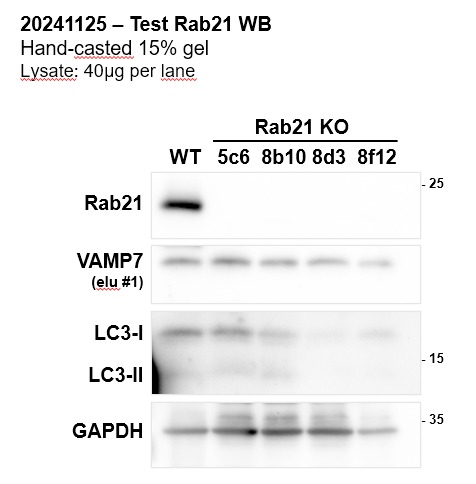


**Rab21-**

**GAPDH-**

**WT**

**KO**

**#1**

**KO**

**#2**

**KO**

**#3**

**KO**

**#4**

**-25**

**-35**

**H)**


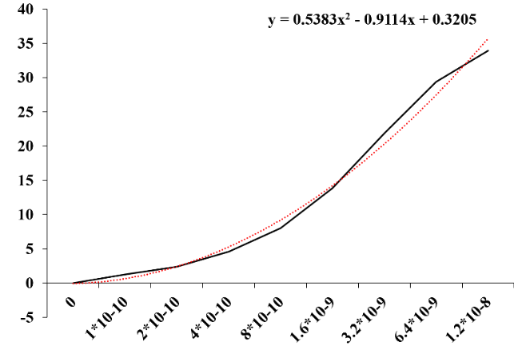


**Relative Light Units (R.L.U.)**

**Concentration (M)**


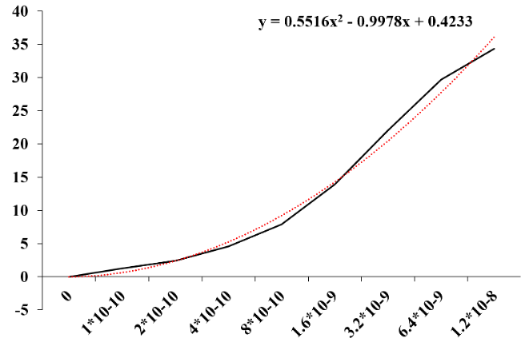


**Relative Light Units (R.L.U.)**

**Concentration (M)**


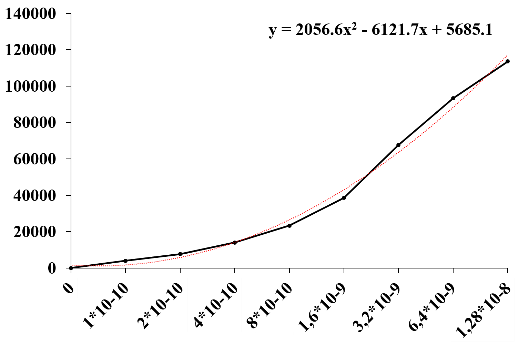


**Arbitrary Units (A.U.)**

**Concentration (M)**

**Figure S1.** Extracellular ATP is diminished in VAMP7, VARP and RAB21 KO HeLa cells as well as in RAB21^T33N^ overexpressing cells. **(A)** Western Blotting of WT and VAMP7 KO HeLa cells generated (VAMP7 KO#1 was previously published). Monoclonal antibody was used to detect VAMP7 depletion (TG49). **(B)** Western Blotting of WT and VARP KO HeLa cells generated. Monoclonal antibody was used to detect VARP depletion (TG40a). GAPDH was used as charge control. **(C)** ATP calibration curve used to obtain the polynomial equation to get the extracellular ATP concentration. Luminescence was measured in Relative Light Units (R.L.U.). **(D)** Quantification of extracellular ATP concentration of HeLa Scramble or VAMP7 KO cells after incubation in control or starvation conditions for 2 hours. **(E)** Quantification of extracellular ATP concentration of HeLa Scramble or VARP KO cells after incubation in control or starvation conditions for 2 hours. **(F)** Western Blotting of WT and RAB21 KO HeLa cells generated Monoclonal antibody was used to detect RAB21 depletion. GAPDH was used as charge control. **(G)** ATP calibration curve used to obtain the polynomial equation to get the extracellular ATP concentration. Luminescence was measured in Relative Light Units (R.L.U.). **(H)** Quantification of extracellular ATP concentration of HeLa Scramble or RAB21 KO cells after incubation in control or starvation conditions for 2 hours. **(I)** ATP calibration curve used to obtain the polynomial equation to get the extracellular ATP concentration. Luminescence was measured in Arbitrary Units (A.U.). **(J)** Quantification of extracellular ATP concentration of HeLa cells overexpressing GFP, GFP-RAB21 WT or GFP-RAB21^T33N^ and incubated in control or starvation conditions for 2 hours. Statistical analyses were done using raw or transformed data of at least three independent experiments and a 2-way ANOVA was performed. In cases were normality was not reached, non-parametric tests were used. Significance: * p≤0.05; ** p ≤ 0.01; *** p≤0.001 and **** p ≤0.0001.


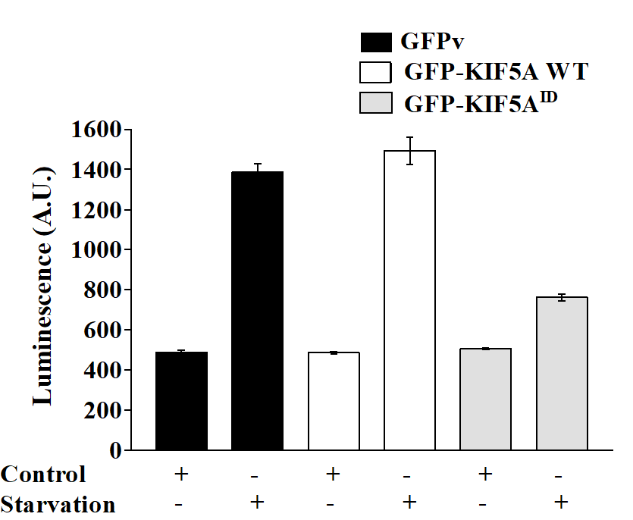


**B)**

**A)**


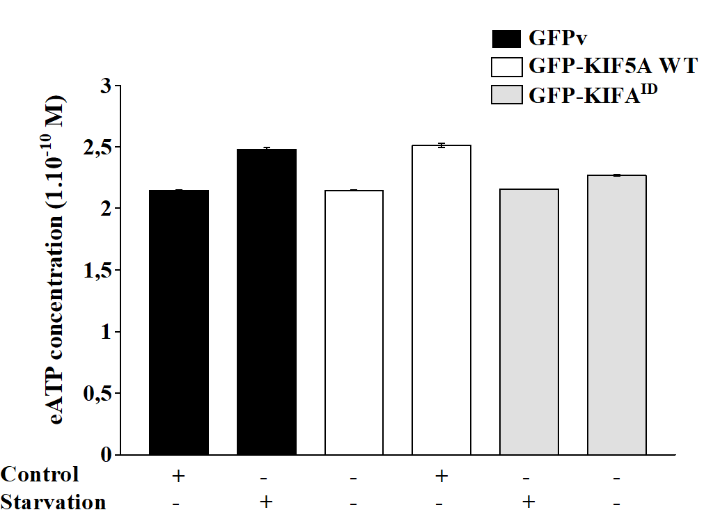


**Figure S2**. KIF5A could participate in ATP autophagy-dependent exocytosis in HeLa cells. (**A**) Measurements (in A.U.) of Luciferin-Luciferase assay of HeLa cells overexpressing GFP, GFP-KIF5A WT or GFP-KIF5A interacting domain (ID) and incubated in control or starvation conditions for 2 hours. (**B**) Extracellular ATP concentration obtained using the ATP calibration curve of Fig. S1i. Results are shown by Mean ± SEM. No statistical differences were found.


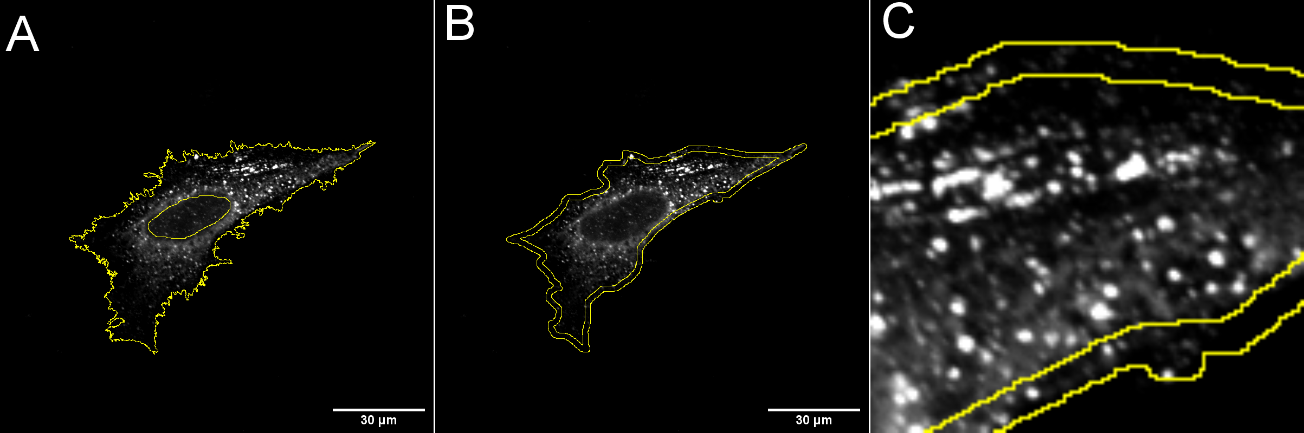


**Figure S3**. Regions Of Interest (ROIs) used in this article. (**A**) Whole cell quantifications were made by using the “wand tool” of ImageJ, with the addition of cutting off the nucleus in order to quantify only the cytoplasm. (**B**) Periphery ROI selection made by using the Selection Brush Tool of ImageJ. The space inside each ROI has a 10 pixels width. (**C**) Magnification of cell periphery where some vesicles can be appreciated inside the ROI. Scale bar: 30 µm.


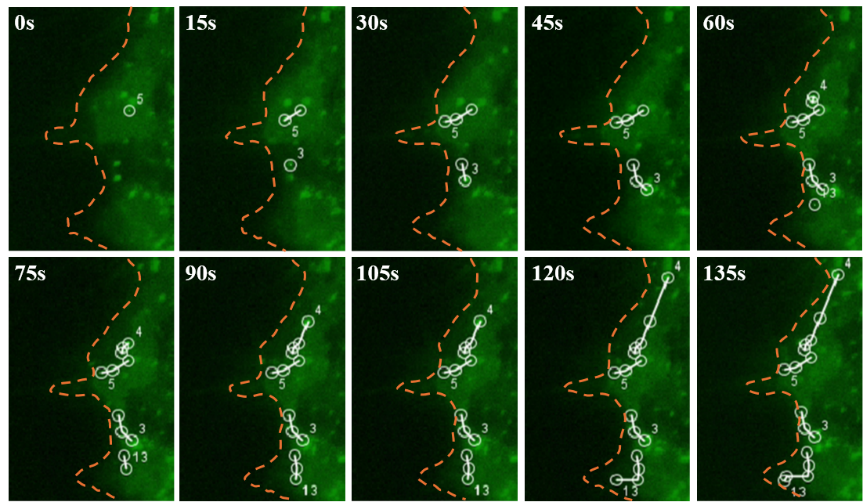

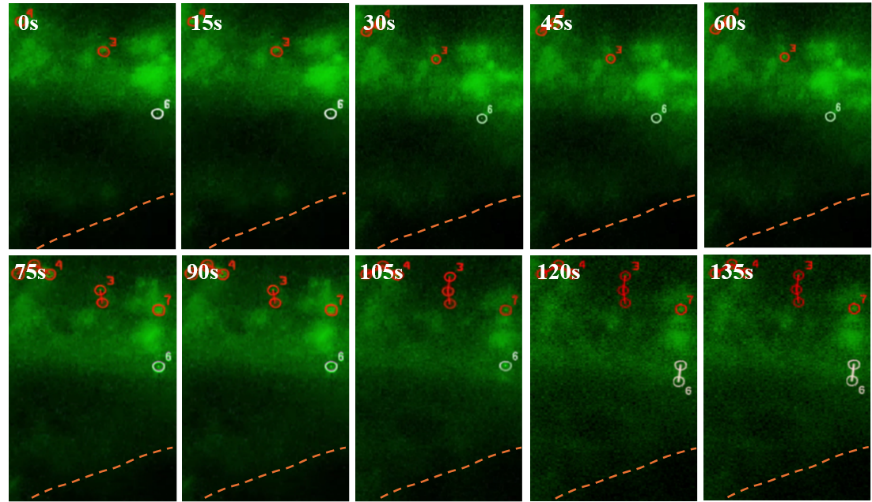


**A)**

**B)**

**C)**


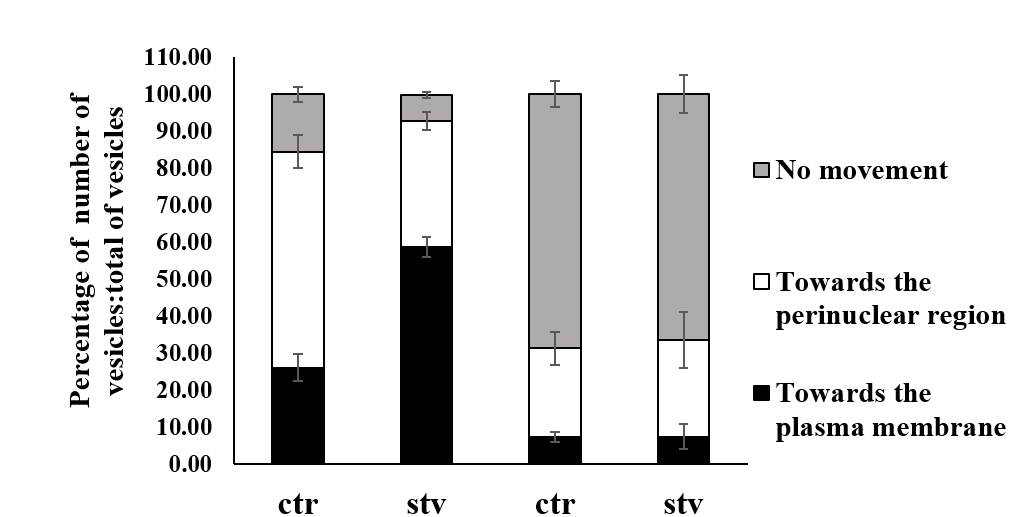


**RAB21 WT**

**RAB21^T33N^**

GFP-RAB21^T33N^

GFP-RAB21 WT

**Figure S4**. RAB21 WT overexpression leads to a vesicle movement towards the plasma membrane in starved HeLa cells. Representative images of 10-minutes time lapse of one cell overexpressing GFP-RAB21 WT (**A**) or GFP-RAB21^T33N^ (**B**) after 2 h starvation treatment. (C) Quantification of the percentage of vesicles per cell that moves towards the cell periphery, the perinuclear region or that shows no movement during the time-lapse. Results are shown by Mean ± SEM. References: ctr: control; stv: starvation.


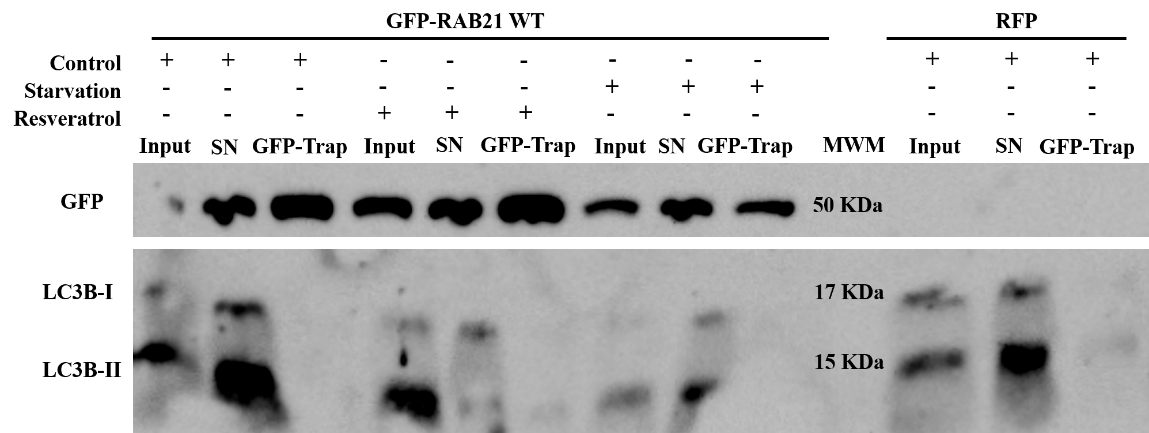


**Figure S5**. RAB21 did not interact directly with LC3B. HeLa cells overexpressing GFP-RAB21 WT or RFP were incubated in control, 50 µM Resveratrol or starvation conditions for 2 or 4 hours and then samples were collected for a GFP-Trap assay followed of 15% SDS-PAGE and Western Blotting to detect GFP and LC3B. MWM: Molecular Weight Marker.


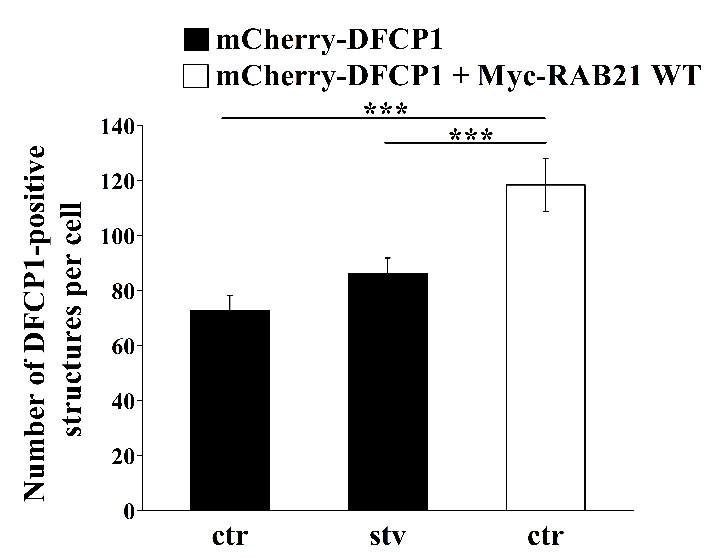

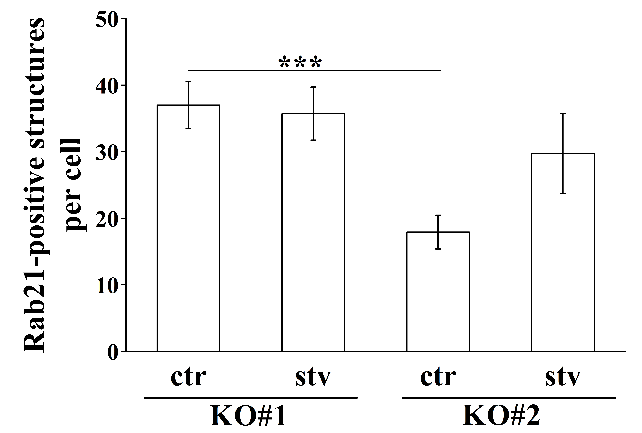

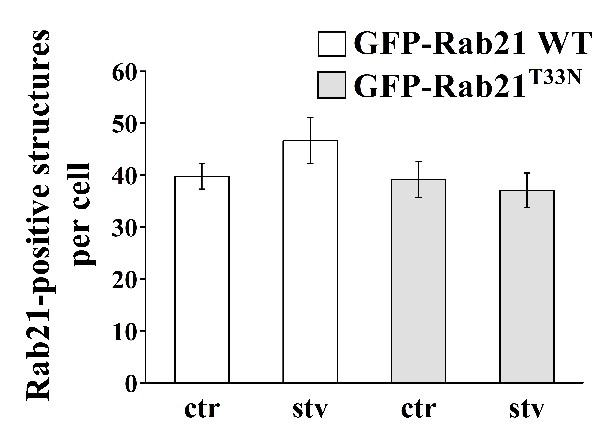


**A)**

**B)**

**C)**

**D)**

**Starvation**

**Myc-RAB21 WT**

**Control**


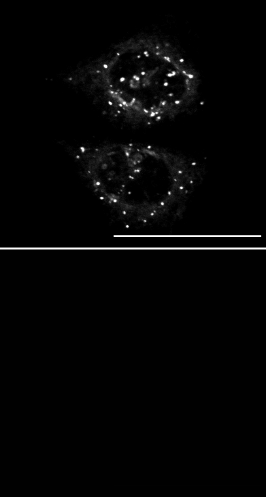

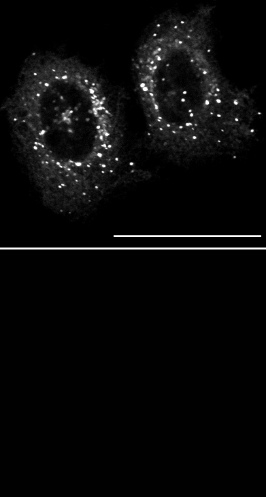

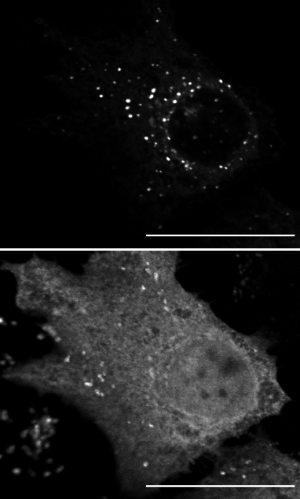


**CFP-DFCP1**

**Myc**

**Figure S6**.  RAB21 WT increased the DFCP1-positive structures at the whole cell in HeLa cells. (**A**) Representative images showing overexpression of CFP-DFCP1 and Myc-RAB21 WT in HeLa cells and then incubated in control (ctr) or starvation (stv) conditions for 2 hours. Scale bar: 30 μm. (**B**) Quantification of DFCP1-positive structures of images shown in (**A**). (**C**) Number of RAB21-positive structures in HeLa cells overexpressing GFP-RAB21 WT or GFP-RAB21^T33N^ and mCherry-DFCP1 after control (ctr) or starvation (stv) treatment for 2 hours (representative images are shown in Figure 6a). No significant differences were found. **(D)** Number of RAB21-positive structures in RAB21 KO cells rescued by overexpression of GFP-RAB21 WT and mCherry-DFCP1 after control or starvation incubation for 2 hours (representative images are shown in Figure 7a). Results are shown by Mean ± SEM. Statistical analyses were done using raw or transformed data of at least two independent experiments and a 2-way ANOVA was performed. In cases were normality was not reached, non-parametric tests were used. Significance: *** p≤0.001.
