# Supplementary material for: A new role of RAB21 and VARP in autophagy and autophagic exocytosis of ATP
Source: Autophagy Rep. 2025 May 11;4(1):2501365. doi: 10.1080/27694127.2025.2501365 (PMC12077462; doi:10.1080/27694127.2025.2501365)
Supplement: Supplemental Material [file KAUO_A_2501365_SM2786.zip › Table S1 MANUSCRIPT.docx]

Table S1: Forward and reverse primers used in this paper to generate VARP and VAMP7 KO HeLa clones.

| Clones | Forward primer | Reverse primer |
| --- | --- | --- |
| VAMP7 1-1 (VAMP7KO#1) and VAMP 1-3 (VAMP7KO#2) | CACCgAACAGCAAAAAGAATCGCCA | AAACTGGCGATTCTTTTTGCTGTTc |
| VAMP7 2-1 (VAMP7KO#3) | CACCgCTTGCCAAACATGCTTGGTG | AAACCACCAAGCATGTTTGGCAAGc |
| VARP 3_1 (VARPKO#1) | CACCGCTCGACAGGCTTCCTTTGC | AAACGCAAAGGAAGCCTGTCGAGC |
| VARP 4_4 (VARPKO#2) and VARP 4_5 (VARPKO#3) | CACCgACTCAAACTGACAAGTAGAC | AAACGTCTACTTGTCAGTTTGAGTc |
